# Physical activity promotion in the early childcare setting: a content analysis of the federal-state-wide educational framework plans in Germany

**DOI:** 10.1186/s12889-025-23798-7

**Published:** 2025-08-14

**Authors:** Stefanie Do, Maike Till, Sven Messing, Peter Gelius, Mirko Brandes, Jonas D. Finger, Sylke Oberwöhrmann, Sarah Forberger, Antje Hebestreit

**Affiliations:** 1 Leibniz Institute for Prevention Research and Epidemiology– BIPS, Bremen, Germany; 2 Department of Sport Science and Sport, Friedrich-Alexander-Universität Erlangen-Nürnberg, Erlangen, Germany; 3 Physical Activity for Health Research Centre, Health Research Institute, Department of Physical Education and Sport Sciences, University of Limerick, Limerick, Ireland; 4 Institute for Sport Science, University of Lausanne, Lausanne, Switzerland; 5 Senate Department for Higher Education and Research, Health and Long-Term Care, Health Department, Berlin, Germany; 6 Department of Health Science, University of York, York, UK

**Keywords:** Early childcare center, Physical activity promotion, Children, Education, Life course, Policy analysis, Germany

## Abstract

**Background:**

Physical activity plays an important role over the life course for non-communicable disease prevention. At an early age, it can support physical, cognitive, and emotional development as well as the establishment of an active lifestyle throughout life. Early childcare centers provide a valuable opportunity in promoting physical activity. Yet, it is unclear, whether and to what extent policy documents such as educational framework plans incorporate physical activity promotion activities and structures in the early childcare setting. We aim to analyze the integration of physical activity in educational framework plans in Germany.

**Methods:**

Ten criteria were identified based on a policy document on prevention indicators and an analytical framework for effective physical activity policy measures: (1) Physical activity as a topic in the educational framework plan, (2) availability of a pedagogical concept for physical activity, (3) objectives, (4) target groups, (5) timeframe, (6) budget, (7) implementation plan, (8) stakeholder involvement across political sectors, (9) legal status, and (10) policy evaluation. Information on the criteria was obtained from the educational framework plans, further policy documents, and a standardized self-administered questionnaire.

**Results:**

All educational framework plans addressed physical activity in a subchapter, while none provided a pedagogical concept on its implementation. The objectives focused on competence goals and related policy measures for physical activity promotion. Most of the educational framework plans targeted children up to 6 years and considered different subgroups. While a timeframe and budget were not mentioned, concrete assistance for policy implementation was provided. On average, three sectors were involved in the educational framework plans’ development. The implementation was binding for 12 out of 16 educational framework plans. Half of the educational framework plans were evaluated either internally or externally.

**Conclusions:**

Educational framework plans describe physical activity promotion for the early childcare setting at a federal state level through their focus on children’s competence development. However, minimum criteria for the implementation and evaluation of PA promotion measures are lacking in educational framework plans. Further research is needed to explore the role of educational framework plans in health promotion, considering the heterogeneity across federal states.

**Trial registration:**

Not applicable.

**Supplementary Information:**

The online version contains supplementary material available at 10.1186/s12889-025-23798-7.

## Introduction

Non-communicable diseases (NCDs) contribute up to 75% of the world’s deaths and are driven by many unhealthy behaviors, such as physical inactivity and excessive sedentary times, that start in childhood and are maintained into adulthood [1, 2]. Physical inactivity is among the leading risk factors for death and disability in the WHO European Region [3]. Recent data suggests that globally 31% of adults [4] and 81% of adolescents [5] are physically inactive [6].

Physical activity (PA) plays an important role at an early age which is marked by rapid physical, cognitive, and emotional development at birth [7]. Young children have a higher need to exercise which is rooted in ancient survival but decreases over the life course below the minimum level necessary for a healthy life [8]. Thus, the early life stage presents an important period for developing healthy behaviors that can promote an active lifestyle, and shape PA levels and patterns throughout the life course [9, 10]. In 2018, the World Health Organization (WHO) launched guidelines on PA, sedentary behavior, and sleep for children under five years of age [11]: Children should engage in at least 180 min of PA per day, of which, starting from the age of three, at least 60 min should be of moderate to vigorous intensity. While previous studies found positive effects in achieving these guidelines on weight status, bone health, cardiometabolic health, and cognition in children under the age of six [12–14], other studies indicate that these recommendations were not met by many countries [15]. For instance, data from Germany reveal that only 42.5% of girls and 48.9% of boys aged three to six years were active for at least 60 min a day; with numbers being drastically lower among 11- to 17-year-olds [16, 17]. As most children and adolescents in Germany do not meet the global PA recommendations [18], PA promotion in early life becomes imperative. The socio-ecological model provides a theoretical framework for implementing effective health promotion strategies by targeting different levels of health determinants from the individual to the systems level [19, 20]. Supportive environments at the organizational level, for instance, can provide opportunities for structured and unstructured PA and, thus, contribute to the cognitive and physical development in early life [10, 11]. Early childcare centers (ECCs) provide a unique setting to promote PA among children [11]. A recent systematic review investigating the effectiveness of international public policies on PA behaviors in preschool children found positive effects of policies implementing PA into the curriculum, requiring a mandatory PA time of at least 60 min per day or revising accreditation standards on the PA environment. Yet, due to different methodologies used in previous studies, results remain inconclusive regarding health outcomes observed in children [21].

In Germany, PA promotion programs in ECCs are regulated at the federal state level [22] which can lead to different approaches in implementation due to the independence of the federal states. 92% of children from the age of three attend ECCs [23] which follow federal-state specific policies described in educational framework plans (EFPs). These policy documents aim to improve the quality of education offered in ECCs by determining the educational mission, defining educational areas, and describing requirements to achieve educational goals [22]. While a large body of research generated evidence on effective interventions for PA promotion in ECCs [21, 24, 25], less is known about how PA promotion is described in policy documents for ECCs, especially in the national context. We hypothesize that policy documents for ECCs in Germany provide guidance on PA promotion in this setting but do not provide detailed information on the implementation and evaluation of PA promotion measures. Therefore, this paper aims at describing how and to which extent PA promotion is included in the EFPs of the 16 German federal states.

## Methods

The mixed-method approach triangulated evidence from different sources by combining content analyses of policy documents with information provided by contact persons in state ministries and senate administrations using a standardized self-administered questionnaire. We included all policy documents related to the development, implementation, and evaluation of EFPs and excluded documents that were not federal-state-specific or published after August 2024. We further excluded information primarily targeting topics such as nutrition and hygiene and addressing PA only as a minor-side aspect. Applying the inclusion and exclusion criteria resulted in a total of 44 documents including EFPs, handouts, guidelines, and evaluation documents. The standardized self-administered questionnaire was sent via e-mail to 16 contact persons in state ministries and senate administrations responsible for the EFPs. Its purpose was to complement information from the policy documents such as the legal status, implementation, and evaluation of EFPs.

An analytical framework was developed to guide content analysis, including ten criteria. Two criteria were derived from a policy document on prevention indicators at state level [26] to guide the content analysis of current EFPs of all 16 German federal states: (1) PA as a topic in the EFP and (2) availability of a pedagogical concept for PA. The remaining criteria were extracted from the overarching analytical framework developed by Daugbjerg et al. [27] which identified effective policy measures for PA promotion. The framework included the following eight criteria: (3) Objectives, (4) target groups, (5) timeframe and (6) budget for policy implementation, (7) implementation plan, (8) stakeholder involvement across political sectors, (9) legal status, and (10) evaluation of policy measures.

Based on this analytical framework, a content analysis was performed on each EFP including further policy documents provided by contact persons in state ministries and senate administrations. PA-specific chapters were selected and distinct search terms (e.g., “physical activity”, “sports”, “exercise”) were identified to ensure a standardized data extraction process in the research team. Information from EFPs of all 16 German federal states was extracted by two independent researchers based on the previously identified ten criteria. Information on the criterion “objectives” was further processed and divided into the two categories “competence development” and “policy measures for PA promotion” in an iterative process. Subcategories for “competence development” were created based on common categorizations in educational research [28]: Self-competence, knowledge competence, social competence, and learning to learn competence. The competence goals were assigned by the research team to each of the competences, if they have not been already categorized in the EFPs [29–38]. Information on the criteria referring to legal status, implementation, and evaluation of EFPs were extracted from the standardized self-administered questionnaire since they were not available in the EFPs.

Table 1 provides an overview of the sources used to derive the information for each of the criteria.

**Table 1:** Sources used for extracting information for physical activity promotion targeting early childcare centers in Germany

Criteria based on Daugjberg et al.^a^ and a German policy document on prevention indicators at state level^b^	Data sources		
	EFPs	Further policy documents^c^	Information derived from standardized self-administered questionnaire^d^
(1) PA as a topic in the EFP	x		
(2) Availability of a pedagogical concept on PA	x		
(3) Objectives^e^	x		
(4) Target groups	x		
(5) Timeframe for the implementation			x
(6) Budget for the implementation			x
(7) Implementation plan	x	x	x
(8) Stakeholder involvement across political sectors	x		
(9) Legal status			x
(10) Evaluation of policy measures		x	x

*Abbreviations*: *EFP* Educational framework plans, *PA* Physical activity

^a^Daugbjerg SB, Kahlmeier S, Racioppi F, Martin-Diener E, Martin B, Oja P, Bull F: Promotion of physical activity in the European region: content analysis of 27 national policy documents. J Phys Act Health 2009, 6(6):805–817

^b^Unterarbeitsgruppe Präventionsindikatoren der Arbeitsgruppe Gesundheitsberichterstattung, Prävention, Rehabilitationund Sozialmedizin (AG GPRS) der Arbeitsgemeinschaft der Obersten Landesgesundheitsbehörden (AOLG). Entwicklung eines Indikatorensystemsfür die Präventionsberichterstattung der Länder. 2021; Accessed from: https://www.bpb.de/themen/bildung/dossier-bildung/292283/bildungsplaene-fuer-kitas/. Accessed 24 May 2025.

^c^E.g., handouts, guidelines, evaluation documents

^d^Derived from answers from state ministries responsible for EFPs

^e^Subcategories: Competence acquisition, measures for promoting PA

## Results

The EFPs differed in terms of length (between 33 and 486 pages) and date of publication (ranging between 2004 and 2024). Their layout greatly varied with some being narratively written, and others being highly structured with, for instance, headings or bullet points. All EFPs followed a similar structure: The first part introduced guiding principles, democratic participation, educational goals, and (basic) competence development of children. Following, the main part covered various educational areas, including natural sciences, languages, arts, or health. The third and last part of the EFPs summarized the requirements for staff, quality development and assurance as well as tasks of the ECC agencies.

The following section describes the information retrieved from the EFPs, further policy documents, and the standardized self-administered questionnaire based on the previously described criteria. Specific EFPs from selected federal states are mentioned when they provide clear examples or when their content deviates from most other EFPs. Table 2 provides a brief overview of each criteria’s data availability in the EFPs. Answers obtained from the standardized self-administered questionnaire are summarized in the supplementary materials for each of the federal states (Additional files 1–3).

**Table 2:** Criteria describing physical activity promotion in federal-state-wide educational framework plans targeting German early childcare centers

Federal State:		BW	BY	BE	BB	HB	HH	HE	MV	NI	NW	RP	SL		SN	ST	SH	TH
1. PA as a topic		✓	✓	✓	✓	✓	✓	✓	✓	✓	✓	✓	✓		✓	✓	✓	✓
2. Availability of a pedagogical concept on PA		✗	✗	✗	✗	✗	✗	✗	✗	✗	✗	✗	✗		✗	✗	✗	✗
3. Objectives	Competences	SEC, KNC, SOC, LEC	SEC, KNC, SOC, LEC	SEC, KNC, SOC, LEC	SEC, KNC, SOC	SEC, KNC, SOC	SEC, KNC, SOC, LEC	SEC, KNC, SOC, LEC	SEC, KNC, SOC	SEC, KNC	SEC, KNC, SOC, LEC	SEC	SEC, KNC, SOC, LEC		SEC, KNC, SOC	SEC, KNC	SEC, KNC, LEC	SEC, KNC, SOC, LEC
	Measures	✗	✓	✓	✓	✓	✓	✗	✗	✓	✓	✓	✓		✓	✓	✓	✓
4. Target groups^a^		0-12^b^	0-10^b^	0–6	0–6	0–6	0–6	0–10	0–10	0–6	0–10	0–10	0–6		0-10^b^	0–6	0–3	0–18

**Federal State**:		**BW**	**BY**	**BE**	**BB**	**HB**	**HH**	**HE**	**MV**	**NI**	**NW**	**RP**	**SL**		**SN**	**ST**	**SH**	**TH**
5. Timeframe for the implementation		Cont.	Cont.	Cont.	Cont.	Cont.	Cont.	Cont.	Cont.	Cont.	Cont.	None		n/a	n/a	None	Cont.	Cont.
6. Budget for the implementation		n/a	n/a	n/a	n/a	n/a	n/a	n/a	n/a	n/a	n/a	n/a	n/a		n/a	n/a	n/a	n/a
7. Implementation plan^c^		ECC agencies	E/F/Y	E/F/Y, UOs of WOs	ECCs	E/F/Y	ECC agencies and UOs	ECC agencies, E/F/Y	ECC agencies, M&S	CY/WO, ECC agencies	ECC agencies	ECC UOs	ECC agencies, E/F/Y		ECC (agencies), M&FS	ECC agencies	ECC agencies, CY/WO	ECC agencies, CY/WO
8. Stakeholder involvement across political sectors^d^		5 (EDU, HEA, OTH, SOA, YOF)	4 (ECO, EDU, OTH, YOF)	3 (EDU, ECO, OTH)	1 (OTH)	4 (EDU, SOA, OTH, YOF)	3 (SOA, OTH, YOF)	3 (ECO, EDU, OTH)	3 (EDU, SOA, OTH)	2 (EDU, OTH)	2 (OTH, YOF)	3 (EDU, OTH, YOF)	3 (EDU, OTH, YOF)		1 (OTH)	3 (EDU, OTH, SOA,)	4 (EDU, OTH, SOA, YOF)	4 (ECO, EDU, SPO, OTH)

**Federal State**:		**BW**	**BY**	**BE**	**BB**	**HB**	**HH**	**HE**	**MV**	**NI**	**NW**	**RP**	**SL**		**SN**	**ST**	**SH**	**TH**
9. Legal status^e^		✓	✗	✓	✓	✓	✓	✗	✓	✗	✓	✓	✓		✓	✓	✗	✓
10. Evaluation of policy measures^f^		✓	✗	✓	(✓)	✗	✗	✓	(✓)	✗	✗	(✓)	✓		✓	✓	✓	(✓)

*Abbreviations*: *BW* Baden-Wuerttemberg, *BY* Bavaria, *BE* Berlin, *BB* Brandenburg, *HB* Bremen, *HH* Hamburg, *HE* Hesse, *MV* Mecklenburg-Western-Pomerania, *NI* Lower Saxony, *NW* North Rhine Westphalia, *RP* Rhineland Palatinate, *SL* Saarland, *SN* Saxony, *ST* Saxony-Anhalt, *SH* Schleswig-Holstein, *TH* Thuringia, *KNC* Knowledge Competence, *LEC* Learning to learn Competence, *SEC* Self-Competence, *SOC* Social Competence, *PA* Physical activity, *C* Continuously, *CY/WO* Child and youth welfare organizations, *ECC* Early childcare center, *E/F/Y* State ministry of education, family or youth, *M&FS* Municipality and federal state, *M&S* Management and staff, *UO* Umbrella organization, *ECO* Economics, *EDU* Education, *HEA* Health, *OTH* Other, *SOA* Social affairs, *SPO* Sports, *YOF* Youth/Family

^a^Age range of children in years

^b^Considers also families, gender, children living with disability or chronic illnesses, or children from disadvantaged backgrounds

^c^Who is responsible for the implementation of the educational framework plan?

^d^Number of political sectors

^e^Is the educational framework plan a binding document?

^f^Is the implementation of the education framework plan being evaluated?

Legend: ✓: Yes, ✗: No, (✓): Partly, n/a: Information not available

1. PA as a topic in the EFP
  All EFPs addressed the topic of PA in subchapters, whose length varied from two to 29 pages. The subchapters represented one of the educational areas, which included the terms ‘body’, ‘PA’ and ‘health’ or related ones.
2. Availability of a pedagogical concept on PA
  None of the EFPs mentioned a pedagogical concept on PA.
3. Objectives
  All EFPs included the promotion of PA with a focus on developing children’s competencies. Competence development was typically accompanied by practical examples or related policy measures and divided into different competence goals. *Self-competence* was highlighted in all EFPs with objectives like fostering a positive relationship with one’s body, developing a natural desire for PA, or experiencing personal movement-related limits. Except for one EFP [34], all EFPs included objectives for *knowledge competence* which encompassed the development of physical fitness, coordination, as well as motor skills and abilities including the use of play equipment, and various materials and terrain. A smaller proportion of EFPs listed objectives for the *social competence* [30, 31, 33, 35, 38–42] focusing on fair cooperation in joint play or engaging with others through movement. About half of the EFPs [29, 33, 37–39, 41–44] outlined objectives for the *learning to learn competence*, which, for instance, included learning that repetition and practice are methods to improve physical skills [42, 44]. Next to the competence goals, one EFP also described health-related goals that included the compensation of physical inactivity and the strengthening of posture [43]. A detailed overview of all competence goals can be found in the supplementary materials (Additional file 4).
  Recommended policy measures for PA promotion described in the EFPs can be divided into measures for the *structural* and *social environment*. Those on the structural environment focused on the *design of indoor/outdoor areas* with general recommendations provided in nine EFPs [30–36, 38, 44], and more detailed examples in four EFPs [37, 41–43]. These included, the availability of a gym or multipurpose-room that can be freely accessed every day for indoor areas [43] and an activity-friendly design with places for climbing, jumping or slopes for outdoor areas [42]. General recommendations on play equipment were available in three EFPs [31, 33, 37] and detailed recommendations and practical examples in seven EFPs [30–32, 38, 41, 43, 44]. Suggested play equipment and materials included skateboards, trampolines, ropes, balls, climbing walls, balancing opportunities or vehicles [41]. Regarding the social environment, most EFPs addressed the role of pedagogical staff [30–32, 34, 37, 41–44], such as staff acting as role models for the joy of movement and healthy activity [44], with three EFPs providing more in-depth suggestions for pedagogical staff [30, 36, 38]. Some EFPs included recommendations for parental involvement [36, 43, 44] or the cooperation with other organizations such as sports clubs and specialist services [34, 36, 43]. General pedagogical suggestions for the promotion of PA were included in 12 EFPs [30–36, 38, 41–44], such as including movement as a direct and indirect goal in other educational areas (somatic, communicative, aesthetic and scientific education) [35]. Additionally, some EFPs provided specific examples for exercise and PA [30–33, 37, 38, 41, 42, 44], with one EFP providing more detailed recommendations [43]. Examples include implementing movement through ball games, running and catching games, skill and reaction games [37].
  Table 3 provides a summary of the policy measures for PA promotion. A more comprehensive overview with examples is provided in the supplementary materials (Additional file 5).

**Table 3:** Measures for physical activity promotion in German early childcare centers at federal state level

Federal state:		BW	BY	BE	BB	HB	HH	HE	MV	NI	NW	RP	SL	SN	ST	SH	TH
**Structural environment**	Recommendations for design of indoor/outdoor areas	-	++	++	+	+	+	-	-	+	+	+	++	+	+	++	+
	Recommendations for play equipment	-	++	++	++	+	++	-	-	++	+	-	++	-	-	+	++
**Social environment**	Recommendations for staff	-	+	++	+	+	+	-	-	+	-	+	+	-	++	+	++
	Recommendations for parental involvement	-	+	-	-	-	+	-	-	-	-	-	-	-	+	-	-
	Recommendations for cooperating with other organizations	-	+	-	-	-	-	-	-	-	-	+	-	-	+	-	-
	General pedagogical suggestions for the promotion of physical activity	**-**	+	+	+	+	+	-	-	+	+	+	+	+	+	-	+
	Specific exercise/physical activity recommendations (e.g. games or exercise-lessons)	-	++	+	+	+	+	-	-	+	+	-	+	-	-	+	+

*Abbreviations*: *BW* Baden-Wuerttemberg, *BY* Bavaria, *BE* Berlin, *BB* Brandenburg, *HB* Bremen, *HH* Hamburg, *HE* Hesse, *MV* Mecklenburg-Western-Pomerania, *NI* Lower Saxony, *NW* North Rhine Westphalia, *RP* Rhineland Palatinate, *SL* Saarland, *SN* Saxony, *ST* Saxony-Anhalt, *SH* Schleswig-Holstein, *TH* Thuringia

Legend: - not mentioned; + mentioned; ++ mentioned with detailed description

4. Target groups
  Most EFPs addressed age groups between 0 and 6 [31, 32, 36, 41, 42, 44] and 0–10 years [33–35, 39, 40, 43]; one EFP covered the age range up to 18 years [38]. Some EFPs also targeted the children’s families [43] or considered gender differences [29, 35], children living with disability [29] or with chronic illnesses [29] or children from disadvantaged backgrounds [35].

5. Timeframe for the implementation
  No fixed timeframe was mentioned for achieving the objectives. All federal states worked toward them on a continuous basis.
6. Budget for the implementation
  No budget was mentioned in any of the EFPs.
7. Implementation plan
  Concrete assistance for implementing the policy measures for PA promotion was predominantly formulated in the EFPs as well as in handouts and guidelines. These recommendations could be divided into practical examples or suggestions for ECCs. In most of the federal states [29, 32, 33, 35–41, 44], the responsibility for implementing the political measures, as outlined in the EFPs, laid with the ECC agencies and other stakeholders, e.g., state ministries for education, family, or youth, child and youth welfare organizations, umbrella organizations of welfare organizations or the ECC management and staff.
8. Stakeholder involvement across sectors
  On average, stakeholders from three sectors were involved in the development of the EFPs. The involved sectors included: Economics, education, health, social affairs, sports, youth/family, and “other”. All EFPs reported involving stakeholders from the “other” sector who could not be assigned to one of the established sectors. The “other” sector comprised of stakeholders such as the municipal council [29, 32, 34, 39, 41, 43], scientific institutions [29–31, 33, 35–37, 39, 40, 42–44], non-governmental organizations [31, 38, 40, 44], welfare and their umbrella organizations [29–32, 34, 37, 38, 40, 41, 43, 44], representatives of people with migrant background [38] or representatives from the church [29, 31, 32, 38, 41]. Eleven EFPs included stakeholders from the education sector [29, 31, 32, 34, 35, 37–43] covering the federal ministry of education, state ministries or institutions in the fields of education/culture, parents’, teachers’ or students’ council, ECC agencies, supervision or professionals. Eight EFPs involved the youth/family sector [29, 31, 33, 34, 37, 41, 43, 44] with the state ministry or the youth welfare offices, while six EFPs included the social affairs sector [29, 31, 36, 37, 40, 44] with state ministries. Four EFPs mentioned stakeholders from the economics sector with commerce or industry related organizations [38, 39, 42, 43]. Only one EFP included the health sector with the state ministry [29], while another one involved the sports sector with the state ministry [38].
9. Legal status
  The implementation for 12 out of 16 EFPs [29, 30, 33–36, 38, 40–42, 44] was binding for the federal state government, subordinate authorities, and ECCs. However, the level of enforcement varied, ranging from voluntary commitment to unspecified sanctions. It was not clearly stated who was responsible for overseeing compliance with these commitments.
10. Evaluation of policy measures
  Based on the information received from the standardized self-administered questionnaires, about half of the federal states assessed the implementation of the EFPs via different evaluation forms. On the one hand, these included internal evaluations in which the ECCs [34] evaluated themselves or together with the parents’ council and children [38]. Internal evaluation documents were not publicly available. On the other hand, external evaluations were conducted by the ECC agencies [34], or local youth welfare organizations [30], by research institutes focusing on health [40] or child and youth research [29, 39, 42], or the state ministry responsible for the EFPs [35, 37]. For one EFP [36], the responsible institution for the external evaluation was unknown since it was evaluated at the time of this publication. Previous external evaluations included impact measures conducted for all educational goals described in the EFPs and did not specifically target the policy measures for PA promotion. Documents from external evaluations were only partly available from the responsible institutions upon request.

## Discussion

The current mixed-methods study triangulated evidence from policy documents to describe how and to which extent PA promotion in early childhood is included in the EFPs of the 16 German federal states. The information was extracted through pre-defined criteria. Key findings include the gap between EFP content and actual implementation and novel insights on PA promotion at the national level.

The EFPs aimed at setting guidelines with a focus on developing children’s competencies including, for instance, language acquisition and early literacy, motor or socio-emotional development. These guidelines formed the basis for recommendations on the implementation of physical education and PA promoting measures. All EFPs included PA promotion in subchapters and most of them outlined concrete policy measures. Among these policy measures, detailed recommendations were provided for the design of indoor and outdoor areas and the role of the pedagogical staff. However, it is crucial to acknowledge that the sole formulation of recommendations, even if they are detailed, is not sufficient to ensure that these measures are actually implemented, as also indicated by previous research on the effectiveness of PA promotion policies in the ECC setting [21].

On the one hand, the concrete implementation of effective measures was found to depend on the structure of the educational system [45] resulting in different implementation approaches due to the independence of the federal states. Interestingly, countries with a closely related socio-geographical and socio-cultural background like Austria have a federal-state-wide educational framework plan providing guidance on PA for all federal states [46] and a supplement describing more concrete PA promotion measures [47]. Despite lacking the depth of German federal-state-specific EFPs, Austria provides an example on how to harmonize PA promotion measures across federal states.

On the other hand, the implementation process must be framed by appropriate evaluation strategies that not only track the enactment of these measures but also assess their impact on the relevant health outcomes [11]. The implementation of the EFPs was binding in most and evaluated by every other German federal state. These mechanisms support a thorough evaluation of whether the intended changes, such as improvements in PA levels or other health-related outcomes, are being realized. The answers to our standardized self-administered questionnaire revealed that it is neither known who is responsible for overseeing enactment or compliance with the EFPs, nor are consequences specified that follow in case of non-compliance. These results align with the governance structure in Germany in which the local administration is responsible for governance, while ECCs are responsible for the implementation of PA promotion measures. Regarding the internal or external evaluations of the EFPs, it is unclear how results are disseminated among stakeholders. Without robust and transparent evaluation mechanisms and communication of outcomes, it remains challenging to monitor progress or identify areas that require further refinement to achieve positive health outcomes.

This study provides novel insights on PA promotion at the national level. While most policies for PA promotion have been investigated at the school level [48, 49] or within a single state [50], this study contributes to further understanding of the ECC setting and highlights the need for a comprehensive, setting-based approach that actively involves the health, sports, and education sectors [3, 51]. The EFPs may, thus, provide a unique opportunity to enable and foster intersectoral collaboration between the education, health and, sports sectors; yet, stakeholders from the health and sport sectors were underrepresented in the design of the EFPs. Fostering intersectoral collaboration may, for instance, include stakeholders from the health and sport sectors being involved in the design and/or evaluation of the EFPs with regards to PA promotion. These collaborations have the potential to create synergies by combining health-related topics, such as promoting PA together with healthy eating [52]. The importance of an intersectoral approach is further highlighted in the WHO global standards for Early Childhood Education and Care Settings, which aim to guide authorities responsible for ECCs in implementing evidence-based recommendations in this setting [53]. By incorporating these recommendations, ECCs can adopt proven strategies that support children’s comprehensive development, while ensuring alignment with global best practices. Given the profound impact of meeting PA recommendations on children’s health [13], it is essential to consider and integrate these guidelines into EFPs to foster a holistic approach to health and learning, and create a win-win situation for the education, health, and sports sectors.

The current study further complements previous findings on the status quo as well as barriers and facilitators of PA promotion in German ECCs [54]. Though two EFPs mentioned children from vulnerable groups and/or with disabilities, they did not specify the inclusion through regulation or fiscal measures. Interestingly, only three EFPs paid special attention to the parental involvement in PA promotion despite parents playing a key role in promoting PA in early life [55] as well as increasing the efficacy of PA promoting measures [45]. Considering that most of the health promotion initiatives in Germany targeted the elementary school level [52] and the current lack of valid data on the PA behavior of children under the age of three [56], this study’s overview offers an additional source for investigating PA promotion in this important age group.

There are several limitations in this study. The mixed-method approach used in this study was tailored to the German federal education system by integrating evidence from federal-state-wide policy documents. Yet, this approach is transferable to other countries which have a similar governance structure for early childhood education. The set of criteria applied in this study to collect information from the EFPs may not have been sufficient enough since EFPs function as guidance documents that do not entail concrete didactical manuals or information on budgets. Yet, this novel approach may be a starting point for future research into the role of EFPs in setting-based health promotion. Data extraction of the policy documents may be subject to different methodological approaches. To reduce a potential bias through different methodological approaches, data were extracted by two independent reviewers and internal meetings were scheduled to facilitate a standardized assessment. The mapping of objectives to competencies was performed by the research team if not provided in the EFPs. Yet, this mapping was based on a scientific framework used in the field of education [28]. Further, comparability between EFPs was not only hampered by federal-state-specific content but also by the time range of about 20 years. Comparing more recent EFPs with older ones, we observed that EFPs became more accessible and shifted towards more concrete description of PA promotion measures. Finally, the EFPs also provided detailed information on other educational areas such as language acquisition and early literacy or socio-emotional development which, depending on the EFP, were discussed either briefly in the PA subchapters or in separate subchapters. However, we limited our research to PA subchapters to keep our focus on the implementation and evaluation of PA promotion measures.

Overall, the strengths of the study lie in the iterative process of triangulating evidence from policy documents and a standardized self-administered questionnaire by an interdisciplinary research team working in the fields of PA, education, child health, and surveillance and monitoring.

## Conclusions

PA is a fundamental prerequisite for physical, cognitive, and emotional development at an early age leading to better child health and shaping better public health in the future by reducing the burden of disease. ECCs provide an opportunity to integrate PA into daily life and establish an active lifestyle throughout life. The EFPs of all 16 German federal states guide ECCs by addressing PA in one of their educational areas and describing the implementation of PA promotion through various criteria. EFPs can provide valuable information on setting-based PA promotion in early childhood at a federal state level through their focus on children’s competence development. Yet, setting minimum criteria for the implementation and evaluation of PA promotion measures in EFPs would allow assessing their effectiveness and additional parameters such as PA engagement. Further investigation is needed to elucidate and improve the role of EFPs in setting-based health promotion.

## Supplementary Information

Additional file 1. Overview of information derived from the standardized self-administered questionnaire (Criterion: Legal obligation)

Additional file 2. Overview of information derived from the standardized self-administered questionnaire (Criterion: Implementation)

Additional file 3. Overview of information derived from the standardized self-administered questionnaire (Criterion: Evaluation)

Additional file 4. Detailed overview of competence development through physical activity promotion

Additional file 5. Detailed overview of political measures for physical activity promotion

## Data Availability

The datasets (educational framework plans) supporting the conclusions of this article are included within the Additional files 1-3 and are available in the repository of the Leibniz Institute for Prevention Research and Epidemiology (BIPS), [https://www.bips-institut.de/fileadmin/downloads/2025-02-23_KAB-Mon_Bildungsrahmenplaene_Metadaten.pdf]

## Abbreviations

ECC: Early childcare center
EFP: Educational framework plan
NCD: Noncommunicable disease
PA: Physical activity

## References

[1] World Health Organization. Fact sheets - Noncommunicable diseases. 2024. Available from: https://www.who.int/news-room/fact-sheets/detail/noncommunicable-diseases. Accessed 24 May 2025.

[2] World Health Organization. Health topics - Noncommunicable diseases. 2025. Available from: https://www.who.int/health-topics/noncommunicable-diseases#tab=tab_1. Accessed 24 May 2025.

[3] World Health Organization. Physical activity strategy for the WHO European Region 2016–2025. 2016. Accessed from: https://www.who.int/europe/publications/i/item/9789289051477. Accessed 24 May 2025.

[4] Strain T, Flaxman S, Guthold R, Semenova E, Cowan M, Riley LM, Bull FC, Stevens GA. National, regional, and global trends in insufficient physical activity among adults from 2000 to 2022: a pooled analysis of 507 population-based surveys with 5.7 million participants. Lancet Global Health. 2024;12(8):e1232–43. 38942042 10.1016/S2214-109X(24)00150-5 PMC11254784

[5] Guthold R, Stevens GA, Riley LM, Bull FC. Global trends in insufficient physical activity among adolescents: a pooled analysis of 298 population-based surveys with 1·6 million participants. Lancet Child Adolesc Health. 2020;4(1):23–35. 31761562 10.1016/S2352-4642(19)30323-2 PMC6919336

[6] World Health Organization. Global action plan on physical activity 2018–2030: more active people for a healthier world. In.; 2018.

[7] Shonkoff JP. From neurons to neighborhoods: old and new challenges for developmental and behavioral pediatrics. J Dev Behav Pediatr. 2003;24(1):70–6. 12584488 10.1097/00004703-200302000-00014

[8] Livingstone MB, Robson PJ, Wallace JM, McKinley MC. How active are we? Levels of routine physical activity in children and adults. Proc Nutr Soc. 2003;62(3):681–701. 14692604 10.1079/PNS2003291

[9] Janz KF, Burns TL, Levy SM. Tracking of activity and sedentary behaviors in childhood: the Iowa bone development study. Am J Prev Med. 2005;29(3):171–8. 16168865 10.1016/j.amepre.2005.06.001

[10] World Health Organization. Improving early childhood development: WHO guideline. 2020. Accessed from: https://www.who.int/publications/i/item/97892400020986. Accessed 24 May 2025. 32200595

[11] World Health Organization. Guidelines on physical activity, sedentary behaviour and sleep for children under 5 years of age. 2019. Accessed from: https://www.who.int/publications/i/item/9789241550536. Accessed 24 May 2025. 31091057

[12] Pate RR, O’Neill JR, Brown WH, Pfeiffer KA, Dowda M, Addy CL. Prevalence of compliance with a new physical activity guideline for Preschool-Age children. Child Obes. 2015;11(4):415–20. 26121562 10.1089/chi.2014.0143 PMC4529021

[13] Börnhorst C, Pigeot I, De Henauw S, Formisano A, Lissner L, Molnár D, Moreno LA, Tornaritis M, Veidebaum T, Vrijkotte T, et al. The effects of hypothetical behavioral interventions on the 13-year incidence of overweight/obesity in children and adolescents. Int J Behav Nutr Phys Act. 2023;20(1):100. 37620898 10.1186/s12966-023-01501-6 PMC10463721

[14] Carson V, Lee E-Y, Hewitt L, Jennings C, Hunter S, Kuzik N, Stearns JA, Unrau SP, Poitras VJ, Gray C, et al. Systematic review of the relationships between physical activity and health indicators in the early years (0–4 years). BMC Public Health. 2017;17(5):854. 29219090 10.1186/s12889-017-4860-0 PMC5753397

[15] Tapia-Serrano MA, Sevil-Serrano J, Sánchez-Miguel PA, López-Gil JF, Tremblay MS, García-Hermoso A. Prevalence of meeting 24-Hour movement guidelines from pre-school to adolescence: A systematic review and meta-analysis including 387,437 participants and 23 countries. J Sport Health Sci. 2022;11(4):427–37. 35066216 10.1016/j.jshs.2022.01.005 PMC9338333

[16] Finger JD, Varnaccia G, Borrmann A, Lange C, Mensink G. Körperliche aktivität von kindern und Jugendlichen in Deutschland–Querschnittergebnisse Aus KIGGS Welle 2 und trends. J Health Mon. 2018;3(1):24–31.

[17] Bucksch J, Möckel J, Kaman A, Sudeck G. Physical activity of older children and adolescents in Germany–Results of the HBSC study 2022 and trends since 2009/10. J Health Mon. 2024;9(1):62. 10.25646/11874 PMC10977466 38559682

[18] Demetriou Y, Beck F, Sturm D, Abu-Omar K, Forberger S, Hebestreit A, Hohmann A, Hülse H, Kläber M, Kobel S, et al. Germany’s 2022 report card on physical activity for children and adolescents. Ger J Exerc Sport Res. 2024;54(2):260–75.

[19] McLeroy KR, Bibeau D, Steckler A, Glanz K. An ecological perspective on health promotion programs. Health Educ Q. 1988;15(4):351–77. 3068205 10.1177/109019818801500401

[20] Dahlgren G, Whitehead M. Policies and strategies to promote social equity in health. Stockholm: Inst Future Stud. 1991;27(1):4–41.

[21] Till M, Volf K, Tristram C, Do S, Gelius P, Hebestreit A, Oberwöhrmann S, Messing S. Evidence on the effectiveness of public policies for physical activity promotion in the early childcare education and care setting: A systematic review. Child Care Health Dev. 2025;51(3):e70078. 40223689 10.1111/cch.70078 PMC11995366

[22] Textor M. Bildungspläne für Kitas. 2019. Accessed from: https://www.bpb.de/themen/bildung/dossier-bildung/292283/bildungsplaene-fuer-kitas/. Accessed 24 May 2025.

[23] Bundesministerium für Familie, Senioren, Frauen und Jugend. Kindertagesbetreuung Kompakt. 2022. Accessed from: https://www.bmbfsfj.bund.de/resource/blob/228470/dc2219705eeb5b8b9c117ce3f7e7bc05/kindertagesbetreuung-kompakt-ausbaustand-und-bedarf-2022-data.pdf. Accessed 24 May 2025.

[24] Lum M, Wolfenden L, Jones J, Grady A, Christian H, Reilly K, Yoong SL. Interventions to Improve Child Physical Activity in the Early Childhood Education and Care Setting: An Umbrella Review. Int J Environ Res Public Health. 2022;19(4):1963. 35206152 10.3390/ijerph19041963 PMC8872396

[25] Trost SG, Ward DS, Senso M. Effects of child care policy and environment on physical activity. Med Sci Sports Exerc. 2010;42(3):520–5. 20068496 10.1249/MSS.0b013e3181cea3ef

[26] Unterarbeitsgruppe Präventionsindikatoren der Arbeitsgruppe Gesundheitsberichterstattung, Prävention, Rehabilitation und Sozialmedizin (AG GPRS) der Arbeitsgemeinschaft der Obersten Landesgesundheitsbehörden (AOLG). Entwicklung eines Indikatorensystems für die Präventionsberichterstattung der Länder. 2021. Accessed from: https://www.bpb.de/themen/bildung/dossier-bildung/292283/bildungsplaene-fuer-kitas/. Accessed 24 May 2025.

[27] Daugbjerg SB, Kahlmeier S, Racioppi F, Martin-Diener E, Martin B, Oja P, Bull F. Promotion of physical activity in the European region: content analysis of 27 National policy documents. J Phys Act Health. 2009;6(6):805–17. 20101924 10.1123/jpah.6.6.805

[28] Weinert FE. Concept of competence: A conceptual clarification. In: Rychen DS, Salganik LH, editors. Defining and selecting key competencies. Seattle: Hogrefe & Huber; 2001. p. 45–65.

[29] Ministerium für Kultus, Jugend und Sport Baden-Württemberg (Hrsg.). Orientierungsplan für Bildung und Erziehung in baden-württembergischen Kindergärten und weiteren Kindertageseinrichtungen. 2011. Accessed from: https://kindergaerten.kultus-bw.de/site/pbs-bw-new/get/documents/KULTUS.Dachmandant/KULTUS/Projekte/kindergaerten-bw/Oplan/Material/KM-KIGA_Orientierungsplan_2011.pdf. Accessed 24 May 2025.

[30] Ministerium für Bildung, Jugend und Sport des Landes Brandenburg (Hrsg.). Bildungsplan – Erweiterte Grundsätze elementarer Bildung in Einrichtungen der Kindertagesbetreuung im Land Brandenburg. 2024. Accessed from: https://mbjs-fachportal.brandenburg.de/kindertagesbetreuung/kita-bildungsplan.html. Accessed 24 May 2025.

[31] Freie Hansestadt Bremen - Die Senatorin für Soziales, Kinder, Jugend und Frauen (Hrsg.). Rahmenplan für Bildung und Erziehung im Elementarbereich. 2012. Accessed from: https://www.bildung.bremen.de/der-bremer-rahmenplan-f-r-bildung-und-erziehung-im-elementarbereich-149916. Accessed 24 May 2025.

[32] Niedersächsisches Kultusministerium (Hrsg.). Orientierungsplan für Bildung und Erziehung im Elementarbereich niedersächsischer Tageseinrichtungen für Kinder. 2023. Accessed from: https://www.mk.niedersachsen.de/download/4491. Accessed 24 May 2025.

[33] Ministerium für Kinder, Familie, Flüchtlinge und Integration des Landes Nordrhein-Westfalen (Hrsg.). Bildungsgrundsätze für Kinder von 0 bis 10 Jahren in Kindertagesbetreuung und Schulen im Primarbereich in Nordrhein-Westfalen. 2018. Accessed from: https://www.kita.nrw.de/system/files/media/document/file/Bildungsgrundsaetze_Stand_2018.pdf. Accessed 24 May 2025.

[34] Ministerium für Bildung, Rheinland-Pfalz (Hrsg.). Bildungs- und Erziehungsempfehlungen für Kindertagesstätten in Rheinland-Pfalz. 2018; Accessed from: https://kita.rlp.de/kita-in-rheinland-pfalz/bildungs-und-erziehungsthemen/bildungs-und-erziehungsempfehlungen-fuer-kindertagesstaetten-in-rheinland-pfalz-plus-qualitaetsempfehlungen. Accessed 24 May 2025.

[35] Sächsisches Staatsministerium für Kultus (Hrsg.). Der sächsische Bildungsplan Der sächsische Bildungsplan - ein Leitfaden für pädagogische Fachkräfte in Krippen, Kindergärten und Horten sowie Kindertagespflege. 2011. Accessed from: https://www.kita-bildungsserver.de/downloads/download-starten/?did=37. Accessed 24 May 2025.

[36] Ministerium für Arbeit und Soziales des Landes Sachsen-Anhalt (Hrsg.). Bildungsprogramm für Kindertageseinrichtungen in Sachsen-Anhalt. Bildung: elementar - Bildung von Anfang an. 2013; Accessed from: https://ms.sachsen-anhalt.de/fileadmin/Bibliothek/Politik_und_Verwaltung/MS/MS/Presse_Dialog_Kita/2014/bildungsprogramm_2014.pdf. Accessed 24 May 2025.

[37] Ministerium für Soziales, Gesundheit, Jugend, Familie und Senioren des Landes Schleswig-Holstein (Hrsg.). Erfolgreich starten - Leitlinien zum Bildungsauftrag von Kindertageseinrichtungen in Schleswig-Holstein. 2020. Accessed from: https://www.schleswig-holstein.de/DE/landesregierung/ministerien-behoerden/VIII/Service/Broschueren/Broschueren_VIII/Kita/BildungsleitlinienDeutsch.html. Accessed 24 May 2025.

[38] Thüringer Ministerium für Bildung, Jugend und Sport (Hrsg.). Thüringer Bildungsplan bis 18 Jahre. 2019. Accessed from: https://bildung.thueringen.de/bildung/bildungsplan/. Accessed 24 May 2025.

[39] Hessisches Ministerium für Arbeit, Integration, Jugend und Soziales (Hrsg.). Bildungs- und Erziehungsplan (BEP) für Kinder von 0 bis 10 Jahren in Hessen. 2019; Accessed from: https://bep.hessen.de/sites/bep.hessen.de/files/2022-11/BEP_2019_Web.pdf. Accessed 24 May 2025.

[40] Ministerium Soziales, Integration und Gleichstellung des Landes Mecklenburg-Vorpommern (Hrsg.). Bildungskonzeption für 0- bis 10-jährige Kinder in Mecklenburg-Vorpommern. 2020. Accessed from: https://www.regierung-mv.de/Landesregierung/bm/Kindertagesfoerderung/Fruehkindliche-Bildung/?id=24717&processor=veroeff. Accessed 24 May 2025.

[41] Ministerium für Bildung und Kultur des Saarlandes (Hrsg.). Bildungsprogramm mit Handreichungen für Saarländische Krippen und Kindergärten. 2018. Accessed from: https://www.saarland.de/SharedDocs/Downloads/DE/mbk/Bildungsserver/Bildungsprogramm/Bildungsprogramm_mit_Handreichungen_zur_Ans. Accessed 24 May 2025.

[42] Senatsverwaltung für Bildung, Jugend und Wissenschaft Berlin (Hrsg.). Berliner Bildungsprogramm für Kitas und Kindertagespflege. 2014. Accessed from: https://www.berlin.de/sen/bildung/schule/bildungswege/fruehkindliche-bildung/. Accessed 24 May 2025.

[43] Bayerisches Staatsministerium für Familie, Arbeit und Soziales & Staatsinstitut für Frühpädagogik (Hrsg.). Der Bayerische Bildungs- und Erziehungsplan (BEP) für Kinder in Tageseinrichtungen bis zur Einschulung. 2019. Accessed from: https://www.ifp.bayern/files/media/ifp/public/books/bildungs-erziehungsplan/index.html. Accessed 24 May 2025.

[44] Freie und Hansestadt Hamburg - Behörde für Arbeit, Soziales, Familie und Integration (Hrsg.). Hamburger Bildungsempfehlungen für die Bildung und Erziehung von Kindern in Tageseinrichtungen. 2012. Accessed from: https://www.hamburg.de/kita/116828/bildungsempfehlungen.html. Accessed 24 May 2025.

[45] Messing S, Rütten A, Abu-Omar K, Ungerer-Röhrich U, Goodwin L, Burlacu I, Gediga G. How Can Physical Activity Be Promoted Among Children and Adolescents? A Systematic Review of Reviews Across Settings. Front Public Health. 2019;7:55. 10.3389/fpubh.2019.00055 PMC6433781 30941342

[46] Charlotte Bühler Institut. Bundesländerübergreifender BildungsRahmenPlan für elementare Bildungseinrichtungen in Österreich. 2019. Accessed from: https://www.bmb.gv.at/Themen/schule/bef/sb/bildungsrahmenplan.html. Accessed 24 May 2025.

[47] Charlotte Bühler Institut. Modul für das letzte Jahr in elementaren Bildungseinrichtungen. Vertiefende Ausführungen zum bundesländerübergreifenden BildungsRahmenPlan. 2019. Accessed from: https://www.bundeskanzleramt.gv.at/dam/jcr:79e29fb6-b02e-47d9-be54-becb7524c060/modul_fuer_das_letzte_jahr_in_elementaren_bildungseinrichtungen.pdf. Accessed 24 May 2025.

[48] World Health Organization. Promoting physical activity in the education sector: current status and success stories from the European Union Member States of the WHO European Region. 2018. Accessed from: https://iris.who.int/handle/10665/345134. Accessed 24 May 2025.

[49] Woods CB, Volf K, Kelly L, Casey B, Gelius P, Messing S, Forberger S, Lakerveld J, Zukowska J, Bengoechea EG. The evidence for the impact of policy on physical activity outcomes within the school setting: A systematic review. J Sport Health Sci. 2021;10(3):263–76. 33482424 10.1016/j.jshs.2021.01.006 PMC8167338

[50] Kohl HW, Johnson AM, Dooley EE, Towner B, Pate RR, Heischmidt K, Elliott EM. An assessment of state-level planning for physical activity promotion in the united States. J Phys Act Health. 2023;20(7):633–8. 37185452 10.1123/jpah.2022-0600

[51] World Health Organization. Multisectoral and intersectoral action for improved health and well-being for all: mapping of the WHO European Region. Governance for a sustainable future: improving health and well-being for all. 2018. Accessed from: https://iris.who.int/handle/10665/341715. Accessed 24 May 2025.

[52] Troppe I, Tanous D, Wirnitzer K. Bewegung & Sport im Lead der schulischen Gesundheitsförderung–Ein systematischer Review der Primarstufen-Curricula in der DA-CH Region im Kontext des Pflichtfächerkanons. Fachzeitschrift Bewegung & Sport, 2, Schwerpunkt Psychosoziale Gesundheit. 2024;78(2).

[53] World Health Organization. Standards for healthy eating, physical activity, sedentary behaviour and sleep in early childhood education and care settings: a toolkit. 2021. Accessed from: https://www.who.int/publications/i/item/9789240032255. Accessed 24 May 2025.

[54] Hermann S, Krug S, Domanska OM, Wurm J, Romefort J, Kuger S, Loss J, Jordan S. Physical activity promotion in daycare centres in germany: study protocol for a cross-sectional survey within the BeweKi study. BMJ Open. 2023;13(6):e070726. 37321809 10.1136/bmjopen-2022-070726 PMC10277102

[55] Petersen TL, Møller LB, Brønd JC, Jepsen R, Grøntved A. Association between parent and child physical activity: a systematic review. Int J Behav Nutr Phys Act. 2020;17:1–16. 32423407 10.1186/s12966-020-00966-z PMC7236180

[56] Messing S, Gelius P, Abu-Omar K, Marzi I, Beck F, Geidl W, Grüne E, Tcymbal A, Reimers AK, Pfeifer K. Developing a policy brief on physical activity promotion for children and adolescents. Front Public Health. 2023;11:1215746. 37841728 10.3389/fpubh.2023.1215746 PMC10571038

