# Supplementary material for: Physical activity promotion in the early childcare setting: a content analysis of the federal-state-wide educational framework plans in Germany
Source: BMC Public Health. 2025 Aug 14;25:2759. doi: 10.1186/s12889-025-23798-7 (PMC12351901; doi:10.1186/s12889-025-23798-7)
Supplement: Supplementary file 1 — Additional file 1. Overview of information derived from the standardized self-administered questionnaire (Criterion: Legal obligation) [file 12889_2025_23798_MOESM1_ESM.pdf]

Additional file 1. Overview of information derived from the standardized self-administered questionnaire (Criterion: Legal obligation)

Federal state	Legal obligation		
	Is the educational framework plan a binding document?	Is there a legal obligation to implement the educational framework plan?	
		If so, how is this liability regulated?	If no, is there any other obligation to implement the educational framework plan (for example, voluntary commitment between the institution and the competent authority) and if so, how is this regulated?
**BADEN-WUERTTEMBERG**	Yes	The orientation plan for education and upbringing in ECCs in Baden-Württemberg and other daycare facilities is binding in its objectives.<br><br>Landesrecht BW KiTaG | Landesnorm Baden-Württemberg | Gesamtausgabe | Gesetz über die Betreuung und Förderung von Kindern in Kindergärten, anderen Tageseinrichtungen und der Kindertagespflege (Kindertagesbetreuungsgesetz - KiTaG) vom 19. März 2009 | gültig ab: 01.01.2009 (landesrecht-bw.de)	**n/a**
**BAVARIA**	No, in the Bavarian educational framework plan the interpretation of the standard is based on the educational objectives set out in the Children's Education Ordinance (AVBayKiBiG). The educational objectives are binding for all state-funded ECCs. However, the implementation of these goals is the responsibility of the providers. The Bavarian educational framework plan serves as a guide.	**n/a**	There is a legal obligation to implement the educational objectives laid down by law. The ECC agencies are responsible for how these are implemented. The Bavarian educational framework plan serves as a guide. Implementation is checked by the supervisory authorities (random sample as part of the document audit).
**BERLIN**	Yes	For systematic quality development and assurance in publicly funded ECCs in Berlin and to anchor the Berlin Education Program for ECCs (BBP) in practice, the state of Berlin has concluded an agreement on quality development in daycare facilities (QVTAG) with the associations belonging to LIGA der Spitzenverbände der freien Wohlfahrtspflege and DaKS e.V., with the participation of the state-owned companies in accordance with § 13 KitaFöG.	**n/a**
**BRANDENBURG**	Yes	The liability is regulated in the KitaG. §3 Abs. 1 KitaG says: The principles agreed under §23, paragraph 3 on the educational work in day care centres form the framework binding for all institutions.	**n/a**
**BREMEN**	Yes	Within the framework of the operating permit by the Landesjugendamt, the concepts of the education plan must be implemented and incorporated into the concepts of the agencies of the day care centers	**n/a**
**HAMBURG**	Yes	https://www.hamburg.de/fachinformationen/rechtliche-grundlagen/13039922/landesrahmenvertrag-kita/	**n/a**
**HESSE**	No, there is no obligation for ECCs.	**n/a**	Hesse pays additional state funding for ECCs that base their educational work on the principles and principles of the Education and Upbringing Plan for Children aged zero to ten in Hesse (BEP).<br>This is regulated in Section 32 of the Hessian Child and Youth Welfare Act (HKJGB).
**MECKLENBURG-WESTERN-POMERANIA**	Yes	In the KiföG M-V, the binding nature is regulated as follows in § 3 Paragraph 3:<br>“The basis for the individual support of children in child day care is the binding educational concept for 0 to 10-year-old children in Mecklenburg-Vorpommern of the ministry responsible for child day care. The implementation of the educational concept must be reflected in the service, remuneration and quality development agreements in accordance with § 24, taking into account the facility-specific concept.”<br>Section 16(2) of the KiföG M-V also regulates specialist and practical advice as follows “The binding standards of the educational concept for 0 to 10-year-old children in Mecklenburg-Vorpommern apply to specialist and practical advice. In particular, the objectives, content and procedures formulated in §§ 1 and 3 are the subject of the specialist and practical advice.”	**n/a**
**LOWER SAXONY**	The objectives of the orientation plan are to make education work binding and transparent, see introduction.	**n/a**	No, the statutory education and training mandate is regulated in Lower Saxony in §§ 2-4 NKiTaG<br><br>There is a voluntary commitment of the institutions, see preface of the orientation plan.
**NORTH RHINE WESTPHALIA**	Yes	ECC agencies are obliged to draw up a pedagogical concept that is geared towards promoting education in accordance with the educational principles in North Rhine Westphalia. This concept should be comprehensive and inclusive. When applying for an operating license for a daycare facility, providers must submit the educational concept to the responsible state youth welfare office.	The mandatory creation of the pedagogical concept ensures that the implementation of the educational areas anchored in the educational principles is guaranteed.
**RHINELAND PALATINATE**	Yes	With the signature of the 2004 Education and Education Recommendations for Day Care Centres in Rhineland-Palatinate (BEE), the Ministry responsible for education, the representation of the Protestant churches, the Catholic dioceses, the LEAGUE of the leading associations of free welfare care and the municipal leading associations, "to support the implementation of education and training recommendations on the basis of their possibilities and taking into account the respective structures."<br><br>The recommendations for education and training are thus jointly developed by all and are accompanied and maintained in their implementation in a joint responsibility for the care system. They are the basis for the design of each ECC.<br><br>With the validity of the new KiTaG from 01.07.2021, the § 24 para. 1 for quality assurance and development that the local public institution of youth assistance with the churches and religious communities of public law, an agreement on the content and quality of education between the associations of independent welfare services at the national level and the local authorities, Education and care in ECCs.	**n/a**
**SAARLAND**	Yes	Ja, Saarländisches Bildungs-, Erziehungs- und Betreuungsgesetz(SBEBG)Vom 19. Januar 2022<br><br>§ 1<br>Basic principles<br>(1) The education, upbringing and care of children in ECCs facilities and in child day care are based on the educational program agreed with the ECC providers with handouts for Saarland nurseries and kindergartens. As part of an inclusive mission, the overall development and unfolding of the child into an independent and socially competent personality should be promoted and the upbringing and education in the family should be supported and supplemented. Parents and guardians are enabled to better reconcile employment and child rearing. A needs-based range of services, particularly with regard to all-day care, serves this purpose. ECCs and child day care are equally important forms of childcare.	**n/a**
**SAXONY**	Yes	Yes, Saxon KiTa Law	**n/a**

**SAXONY-ANHALT**	Yes	Yes, § 5 Para. 3 KiFöG:<br><br>“The ECC agencies are responsible for implementing the educational mandate. The binding basis is the educational program “Bildung: elementar - Bildung von Anfang an” (Education: elementary - education from the beginning) with special attention to language promotion. Each ECC must work according to a concept and a quality management system to be freely chosen by the provider.”<br><br>https://www.landesrecht.sachsen-anhalt.de/bsst/document/jlr-KiF%C3%B6GSTV14P5	**n/a**
**SCHLESWIG-HOLSTEIN**	No – they are recommendations. The educational sectors become binding through Section 19 Paragraph 1 of the KiTaG.	see above (§ 19 Absatz 1 KiTaG)	No, but to implement the educational areas described therein (see above).
**THURINGIA**	Yes	„The educational plan drawn up by the Ministry forms the basis for the educational work in ECCs.“ § 7 Abs. 1 letzter Satz Thüringer Kindergartengesetz (ThürKigaG <https://bildung.thueringen.de/fileadmin/bildung/kindergarten/recht/2023-05-04_ThuerKigaG_Lesefassung_mit_Erlaeuterungen.pdf>)	**n/a**

Legend: n/a: Information not available

Abbreviations: ECC early childcare center; KiföG Child Promotion Act; KitaG Child Day Care Act
