# Supplementary material for: Physical activity promotion in the early childcare setting: a content analysis of the federal-state-wide educational framework plans in Germany
Source: BMC Public Health. 2025 Aug 14;25:2759. doi: 10.1186/s12889-025-23798-7 (PMC12351901; doi:10.1186/s12889-025-23798-7)
Supplement: Supplementary file 2 — Additional file 2. Overview of information derived from the standardized self-administered questionnaire (Criterion: Implementation) [file 12889_2025_23798_MOESM2_ESM.pdf]

Additional file 2. Overview of information derived from the standardized self-administered questionnaire (Criterion: Implementation)

Federal state	Implementation	
	Who is responsible for the implementation of the Education Framework Plan?	What is/was the time frame for the implementation of the objectives?
**BADEN-WUERTTEMBERG**	ECC agencies	Continuously
**BAVARIA**	Bavarian State Ministry for Family, Labour and Social Affairs (Development of the BayBEP: Staatsinstitut für Frühpädagogik und Medienkompetenz (IFP))	The Bavarian educational framework plan was implemented in fall 2005 and has been regularly updated since then, most recently in 2019.<br>A comprehensive revision of the Bavarian educational framework plan is currently being carried out by the IFP (duration still open).
**BERLIN**	The state of Berlin has concluded an agreement on quality development in ECCs (QVTAG) with the associations belonging to the LIGA der Spitzenverbände der freien Wohlfahrtspflege and DaKS e.V. with the participation of its own companies in accordance with Section 13 KitaFöG. The work with the Berlin education program is implemented in joint responsibility between public youth welfare (Land Berlin) and independent youth welfare (LIGA der Wohlfahrtsverbände). Further details can be found in the QVTAG, which I have attached.<br><br>The State of Berlin is responsible for the Berlin Education Program:<br>Senate Department for Education, Youth and Family	In August 2004, the "Berliner Bildungsprogramm für die Bildung, Erziehung und Betreuung von Kindern in Tageseinrichtungen bis zu ihrem Schuleintritt" was published as a professional framework for all ECCs and made available to all Berlin ECCs. The "Vereinbarung über die Qualitätsentwicklung in Berliner Kindertagesstätten – Qualitätsvereinbarung Tageseinrichtungen – QVTAG", which was concluded with all provider associations on 12.01.2006 and updated in 2020, has made working with the aforementioned educational program a binding professional basis for all publicly funded ECCs in Berlin.<br><br>This first version of the education program was updated in 2014 ("Berlin's Early Years Programme for early years centres and family day-care" (BBP)) and expanded to include child day care in particular. Furthermore, greater importance was placed on inclusive education and the integration and early support of children with additional support needs. After 10 years, the BBP is currently undergoing further development and is to be introduced for the 2024/25 nursery year. Priorities currently being updated have been identified on the basis of new scientific findings and developed together with practitioners in a multi-year participation process. In particular, these include the topics of "Observation, documentation and assessment of child development", "Inclusion and anti-discrimination", "Language, mathematics and digital education", "Health and physical activity" and "Education for sustainable development".
**BRANDENBURG**	According to the KitaG, the institutions are responsible for implementing the principles of elementary education. These describe the implementation of the principles of elementary education and how the quality of pedagogical work is checked (§3 Abs. 3 KitaG).	The implementation of the principles of elementary education takes place continuously
**BREMEN**	Senatorin für Kinder und Bildung, Referat Qualitätsentwicklung und Aufsichtsfunktion in der Kindertagesbetreuung	From 2012 until the introduction of the education plan 0-10 years
**HAMBURG**	Educational recommendations form the binding framework for pedagogical work in all day care centres in Hamburg.<br><br>The implementation and transfer to the educational work of the ECCs is done by the ECC agencies and associations.	**n/a**
**HESSE**	Child day care facilities are responsible for the implementation of the educational framework planand the local providers of public youth welfare services for ECCs.<br><br>Responsible for evaluating and updating the educational framework plan: Hessisches Ministerium für Arbeit, Integration, Jugend und Soziales and Hessische Ministerium für Kultus, Bildung und Chancen	The measures for implementing the educational framework plan in Hesse are designed for long-term implementation. The Hessian Child and Youth Welfare Act as the regulating state law is currently valid until December 31, 2025.
**MECKLENBURG-WESTERN-POMERANIA**	In KiföG M-V, § 10 Paragraph 2 regulates implementation as follows:<br>"The provider of the child day care facility shall draw up a binding educational concept for the child day care facility, which describes and specifies the implementation of the educational concept for 0 to 10-year-old children in Mecklenburg-Vorpommern and the objectives and tasks listed in §§ 1 and 3. The educational concept is to be updated on an ongoing basis."<br>The KiföG M-V also regulates the topic of training and further education in § 17 Paragraph 3:<br>"The local providers of public youth welfare services must provide or arrange sufficient needs-oriented further training and advisory services based on the objectives and content of the educational concept for 0 to 10-year-old children in Mecklenburg-Vorpommern for the educational professionals, insofar as this is not done by the ECC agencies or their respective umbrella or umbrella organizations themselves."<br><br>There is no central institution or organization, as the providers themselves are responsible for implementation. Accordingly, no specific contact details can be provided.	The implementation of the educational concept is an ongoing process, the binding nature of which is regulated in § 3 Para. 3 KiföG M-V (see above).
**LOWER SAXONY**	The public child and youth welfare institutions as well as other institutions of ECCs.	Continuously
**NORTH RHINE WESTPHALIA**	The implementation of the educational principles in North Rhine Westphalia is primarily the responsibility of the providers, management and educational staff.	Continuously
**RHINELAND PALATINATE**	Each of the signatory ECC umbrella organizations is responsible for "to support the implementation of education and training recommendations on the basis of their possibilities and taking into account the respective structures.<br>The Ministry of Education can act as a contact point.	The validity began in 2004 and provides a framework for the process-related quality improvement without any maximum validity period being specified. A revision of the educational framework plan is planned.
**SAARLAND**	The ECC managers, Inspection by the Ministry of Education and Culture	Not defined
**SAXONY**	Agencies/Institution; municipal self-administration, country is only partially responsible	Not given
**SAXONY-ANHALT**	§ 5 Para. 3 KiföG:<br>"The ECC agencies are responsible for implementing the educational mandate. The binding basis is the educational program "Bildung: elementar - Bildung von Anfang an" (Education: elementary - education from the beginning) with special attention to language promotion. Each ECC must work according to a concept and a quality management system to be freely chosen by the provider."<br><br>https://www.landesrecht.sachsen-anhalt.de/bsst/document/jlr-KiF%C3%B6GSTV14P5	The KiföG applies. There is no time frame for the implementation of objectives.
**SCHLESWIG-HOLSTEIN**	The facility operators are responsible for implementation. The local public youth welfare agency is responsible for monitoring compliance with daycare quality as a funding requirement in accordance with Section 35 of the KiTaG. The state is responsible for the educational guidelines themselves as a recommendation for the KiTaG.	Continuously and unlimited
**THURINGIA**	The Thuringian Education Plan is implemented in all Thuringian ECCs. „Each ECC creates a compulsory pedagogical concept for it, which describes and specifies the implementation of the objectives and tasks set out in the education plan and in paragraphs 1 to 3. The concept contains statements on the design of educational, educative and care processes in consideration of a healthy lifestyle as well as the pedagogical space design. The pedagogical concept is continuously continued " See § 7 Paragraph 4 ThürKigaG.<br><br>The Thuringian Ministry for Education, Youth and Sport is responsible for supervising ECCs.The examination also assesses the design and implementation of the educational framework plan.	The Thuringian education plan for children up to 18 years of age is fully implemented in all ECCs in Thuringia. The implementation is carried out individually and according to requirements in the facilities within the framework of the always-on-trend design. In addition to the review by the supervisory authority, the audit is carried out in accordance with § 7 para. 7 ThürKigaG a continuous self-evaluation with the involvement of the parents' council and the children.

Legend: n/a: Information not available

Abbreviations: ECC Early childcare center; KiföG Child Promotion Act; KitaG Child Day Care Act
