# Supplementary material for: Physical activity promotion in the early childcare setting: a content analysis of the federal-state-wide educational framework plans in Germany
Source: BMC Public Health. 2025 Aug 14;25:2759. doi: 10.1186/s12889-025-23798-7 (PMC12351901; doi:10.1186/s12889-025-23798-7)
Supplement: Supplementary file 3 — Additional file 3. Overview of information derived from the standardized self-administered questionnaire (Criterion: Evaluation) [file 12889_2025_23798_MOESM3_ESM.pdf]

Additional file 3. Overview of information derived from the standardized self-administered questionnaire (Criterion: Evaluation)

Federal state	Evaluation		
	Is the implementation of the educational framework plan being evaluated?	*If yes* , who is the responsible contact for evaluation?	What criteria are used for evaluation?
**BADEN-WUERTTEMBERG**	Yes	Evaluated in 2020/2021 by the Center for Child and Youth Research/ Zentrum für Kinder- und Jugendforschung (ZfKJ), EH Freiburg	Evaluation criteria/results see Evaluation Orientierungsplan (Eval-O BaWü) (zfkj.de): http://www.zfkj.de/index.php/forschungsaktivitaeten/evaluation-orientierungsplan
**BAVARIA**	No	**n/a**	**n/a**
**BERLIN**	Yes	Berliner Kita-Institut für Qualitätsentwicklung (BeKi)	The criteria for external evaluation are described in Annex 2 of the QVTAG as a key points paper for external evaluation with the Berlin education program.<br>Criteria for internal evaluation are described in additional materials from the state of Berlin (toolbox for internal evaluation and collection of materials in the red folder for internal evaluation). Link: https://beki-qualitaet.de/materialien-und-forschungsberichte-uebersicht/materialien-und-forschungsberichte
**BRANDENBURG**	Partially. The local public youth welfare institutions may require ECCs to have their work checked by quality assessments (§3 Abs. 4 KitaG). Many institutions use their own quality development tools.	The local public youth service providers	**n/a**
**BREMEN**	No	**n/a**	**n/a**
**HAMBURG**	No	**n/a**	**n/a**
**HESSE**	Yes	The Staatsinstitut für Frühpädagogik und Medienkompetenz (IFP) in Munich is responsible for the evaluation.	All measures of qualification and process support for the educational framework plan are evaluated with questionnaires for participants and teachers with regard to design, content and impact.
**MECKLENBURG-WESTERN-POMERANIA**	An evaluation is carried out on a pro rata basis as part of targeted individual support (§ 3 Para. 6 KiföG M-V).	University Medicine Greifswald Institute for Community Medicine	The Dortmunder Entwicklungsscreening für den Kindergarten (DESK 3-6 R) is used for the evaluation.
**LOWER SAXONY**	No	**n/a**	**n/a**
**NORTH RHINE WESTPHALIA**	No	**n/a**	**n/a**
**RHINELAND PALATINATE**	Yes and no,<br>Together with the IBEB, ECCs are working on a concept for self-evaluation. In addition, there is the Movement Day Care Centre/ Bewegungskita which evaluates the topic of movement (but it goes beyond the educational framework plan; results are not included in the educational framework plan)	The Education and Training Recommendations are developed jointly by all and are followed up in their implementation in a joint responsibility for the childcare system.	The implementation of the educational recommendations in Rhineland-Palatinate is process-oriented and embedded in the continuous quality development of the individual educational and education areas as well as the individual ECCs. The recommendations are embedded in the quality systems of church institutions. Especially communal day care centers are and were through the approach of quality development in discourse (QiD) of the Institute for Education, Upbringing and Childcare (IBEB) at the University of Koblenz. During this time, the IBEB provides certified support for one year and exchanges information in a regional group.<br><br>https://www.hs-koblenz.de/sozialwissenschaften/institute-des-fachbereichs/institut-fuer-bildung-erziehung-und-betreuung-in-der-kindheit-rheinland-pfalz-ibeb/qualitaetsentwicklung-im-diskurs-qid<br><br>The proven costs of continuing education and specialist advice, inter alia on topics of the educational framework plan but also in the area of quality development will be up to 1% of the other eligible personnel costs included in the framework of the country's personnel cost promotion. In addition, the free agents receive additional allocations to staff costs for quality assurance and development pursuant to § 21, paragraph 1, point 5.
**SAARLAND**	Yes, through specially developed materials for internal evaluation or through external evaluation, ordered by the ECC agencies	The ECC agencies must be asked	Internal evaluation materials, publication of the revision at the end of 2023
**SAXONY**	Yes, continuation is currently taking place	Sächsisches Staatsministerium für Kultus	Still to be determined
**SAXONY-ANHALT**	Yes	The Research and Development Centre (FEZ) was commissioned by the Ministry of Labour, Social Affairs, Health and Gender Equality to further develop the current education programme.<br><br>The implementation is carried out by the Kompetenzzentrum Frühe Bildung der Hochschule Magdeburg-Stendal (KFB).	The education programme „Bildung: elementar – Bildung von Anfang an" will be updated in 2024.<br><br>In this context, the existing educational program is reviewed for its scientific relevance, existing content is adapted and new content is added. The main tasks of the Update are:<br>Analysis and evaluation of the current education program taking into account current scientific knowledge,<br>• Literature research regarding existing or usable scientific studies in, for and about Saxony-Anhalt with the involvement of relevant scientific institutions and Expert/in,<br>• The current educational programme is to be integrated into current socio-pedagogical and educational-scientific discourses and<br>Derivation of important key points for the new version of the educational program,<br>• Expert survey (managers of day care facilities, providers and associations of day care facilities, youth welfare office, youth offices, parents' representatives/ inns) in relation to the current education program (successful factors, difficulties in understanding and implementation) and on technical needs for a content-related updating,<br>• Visits and practical observations in day care facilities,<br>• Reflection on successful practice, identification of stumbling blocks and resulting development processes as well as analysis and use of current scientific surveys at federal and European level.
**SCHLESWIG-HOLSTEIN**	Yes, a first evaluation took place in 2014. A revision of the guidelines is planned for 2024.	State of Schleswig-Holstein, Ministerium für Soziales, Gesundheit, Jugend, Familie und Senioren Schleswig-Holstein.	These were and are determined together with the stakeholders in the system of early childhood education, care and upbringing and the scientific support structure (see imprint/author). Unfortunately, there are currently no comprehensive documents available on the evaluation from 2014.
**THURINGIA**	Yes, in addition to the audit by the supervisory authority, it is carried out in accordance with § 7 para. 7 ThürKigaG a continuous self-evaluation with the involvement of the parents' council and the children.	The ECC supervisor	Internal audit lists are used for the supervisory review and standards communicated to the institutions in accordance with the educational framework plan. The criteria for self-evaluation are usually developed by the institutions themselves in cooperation with the respective institution within the framework of concept development (QM manuals of the ECC agencies).

Legend: n/a: Information not available

Abbreviations: ECC Early childcare center; KiföG Child Promotion Act; KitaG Child Day Care Act
