# Supplementary material for: Physical activity promotion in the early childcare setting: a content analysis of the federal-state-wide educational framework plans in Germany
Source: BMC Public Health. 2025 Aug 14;25:2759. doi: 10.1186/s12889-025-23798-7 (PMC12351901; doi:10.1186/s12889-025-23798-7)
Supplement: Supplementary file 4 — Additional file 4. Detailed overview of competence development through physical activity promotion [file 12889_2025_23798_MOESM4_ESM.pdf]

Additional file 4. Detailed overview of competence development through physical activity promotion

Federal state	Self-competence	Knowledge competence	Social competence	Learning to learn competence
**BADEN-WUERTTEMBERG**[1]	• Children learn basic movements and expand the area in which they act and experience<br>• Children recognize physical needs and respond to them<br>• Children develop a positive body- and self-concept as a base for further development<br>• Children acquire knowledge about their body and increase their body awareness and schema<br>• Children develop an awareness of their own physical abilities and limits, as well as those of others, and learn to accept them.	• Children expand and refine their fine-, grapho- and grossmotor skills and abilities<br>• Children expand their conditional and coordinative skills and abilities	• Children develop an awareness of their own physical abilities and limits, as well as those of others, and learn to accept them.	• Children experience their body as means of representation and and espression for communication, art, music and dance as well as acting and theater.
**BAVARIA**[2]	• Children find a conscious connection to themselves through movement<br>• Children increase their self-esteem through greater movement-confidence<br>• Childen can realistically assess their capabilities<br>• Children experience self-efficacy by independently solving movement tasks<br>• Children maintain joy of movement and their willingness to be active<br>• Children learn to identify individual improvement of performance rather than just comparing themselves to others<br>• Children develop curiosity for new movement patterns and challenging motor skills<br>• Children develop body awareness and bodily consciousness	• Children gain movement experience movement and satisfy elementary movement needs<br>• Children test and refine motor and coordination skills<br>• Children develop conditional skills<br>• Children identify their physical limits and expand them through practice<br>• Children learn how to properly use play objects and sports equipment	•Children develop joy in playing in a group<br>• Children develop a team spirit and learn to cooperate with others in movement tasks<br>• Children experience joy from moving with others<br>• Children learn to understand and adhere to rules<br>• Children practice to be considerate of others, to be fair and to take on responsibility<br>• Children experience movement as form of interaction and communication	• Children learn to concentrate on certain movement patterns<br>• Children learn imagination and creativity by trying out new movement ideas<br>• Children discover problem-solving strategies by dealing with differing movements<br>• Children learn to understand connections between exercise, nutrition and health
**BERLIN**	• Children develop an awareness of what is good for their body and soul and promotes health.<br>• Children learn to perceive and express their own physical, mental, and social needs, interests, and feelings.<br>• Children discover their own body through perception and movement, develop a personal physical identity, and feel comfortable.<br>• Children experience themselves as self-efficacious and want to develop individual strengths<br>• Children experience alternating phases phases of activity/movement and relaxation/rest	• Children can experience a variety of gross- and fine motor movements<br>• Children develop spatial orientation<br>• Children enjoy movement, expand the area in which they are active, and develop flexibility, dexteriity, movement competence and coordination skills<br>• Children become increasingly confident in competitions involving vehicles or other means of transportation, as well as with play and movement materials.<br>• Children learn rules of movement and sports games and can apply them.	• Children enjoy moving with others, inventing their own rules, and learning to recognize the rules of others<br>• Children learn assert their boundaries regarding physical contact and follow rules<br>• Children learn to recognize the physical abilities of others and to help them.<br>• Children learn to engage with or set boundaries around the challenges posed by others.<br>• Children learn to deal appropriately with defeats and victories<br>• Children learn to respect physical diversity and consider others' needs<br>• Children learn to appreciate the athletic achievements of people with disabilities.<br>• Children learn to respect the sports and movement arts of different cultures.	• Children learn that repetition and practice is a method that can improve physical skills<br>• Children develop an awareness of their own abilities and potential for growth<br>• Children learn not to be dissuaded from failures and to try again<br>• Children develop a desire to learn and are curiosity for further experiences and want to expand the area in which they are active
**BRANDENBURG**[1]	• Children learn to regulate and differentiate emotion through movement<br>• Children learn what is good for them and what is not, which behaviors and habits are healthy and what can make them ill<br>• Children learn to perceive their body signals, feel discomfort, recognize their needs, express them verbally, and help to ensure that they are met<br>• Children learn that their positive attitude towards themselves and their body's ability to move is just as important as reliable relationships and support from other people<br>• Children learn how to relax and enjoy themselves and learn what contributes to mental health.<br>'• Children get to know and appreciate their bodies and learn that they have the right to physical self-determination<br>• Children learn to recognize and respect their own boundaries and the boundaries of others and to distinguish appropriate touching from boundary violations or assaults<br>• Children get to know different types of relationships and learn that mutual respect and boundaries are important in all relationships<br>• Children increasingly know about their bodies and name body parts and internal organs<br>• Children are more able to discuss assaults if they can naturally pronounce terms for their body parts, including their genitals	• Children learn to move on inclined surfaces and stepped platforms<br>• Children learn to alternate between fast and slow movements<br>• Children learn to jump or use the swing<br>• Children learn to coordinate their movement to the rhythm of music<br>• Children develop their motor skills, abilities and fitness by spending at least three hours a day with physical activity; at least 60 minutes of which should be at moderate to high intensity; and only sitting for a maximum of 60 minutes at a time.	• In movement games, children learn to cooperate, to set rules for themselves within groups, to follow them and to change them.<br>• Children observe and imitate the movements of others and learn to adapt their movements to a certain rhythm and to the movements of others - such as in dancing or other synchronized movement sequences, and they themselves give impulses that other children take up.<br>• Children learn to master challenging motor tasks together - i.e. to adapt their movements to the requirements and actions of others, to complement and support them. (s.98)<br><br>• Children learn the connection between motor skills, self-esteem and acceptance in the group and experience what is fair and what is unfair.<br>• Children learn about cooperative movement games without winners and losers, in which the main focus is on fun rather than performance and competition.<br>• In competitive games and sporting comparisons, children learn to experience themselves alternately as winners and losers and learn how to deal with victory and superiority, but also with frustration, disappointment and defeat	**n/a**
**BREMEN**[1]	• Children explore their abilities through movement and experience their limits and learn to differenciate between themselves and others<br>• Children can express emotions though their movement and learn that movement can influence their mental state.<br>• Children develop self-awareness and are able to identify bodily needs based on physical activity and relaxation.<br>• Children learn to regulate emotions through movement and recognize its positive effects on mental well-being<br>• Children develop a desire to move and increase their body control and self-confidence as well as develop their social skills and a cognitive understanding<br>• Children develop a positive relationship to their body leads to self-regulation of physical and emotional needs and becomes part of their positive self-perception.	• Children are encouraged in their lateral development<br>• Children develop fine- and graphomotor skills, and dexterity<br>• Children increase their coordination of movement<br>• Children learn to recognize different rhythms and can convert them into sounds and movement	• Children learn to form relationships through movement and to determine the distance or closeness they wish to have with adults and other children<br>• Children learn to cooperate and collaborate with others during physical play	**n/a**
**HAMBURG**	• Children discover their own body through perception and movement<br>• Children learn mobility and dexerity<br>• Children learn movement-confidence and coordination skills<br>• Children learn to be proud of having mastered physical challenges<br>• Children develop an awareness of what is good for their body and soul and promotes health.<br>• Children notice and express their own physical, mental and social needs, interests and feelings, and can communicate them to others while being able to set boundaries<br>• Children experience themselves as self-efficacious and want to develop individual strengths<br>• Children enjoy movement, expand the area in which they are active and experiment with movement<br>• Children learn to advocate their individual boundaries	• Children can point to corresponding body parts, name them when asked and develop a basic understanding of bodily functions<br>• Children develop spatial orientation<br>• Children learn reciprocal right-left grasping and develop handedness<br>• Children can point to corresponding body parts, name them when asked and develop a basic understanding of bodily functions	• Children learn to recognise the physical skills of others and to support them<br>• Children learn to respect physical diversity<br>• Children learn to negotiate rules about desired and undesired physical contact with others, while realistically estimate their own strengths<br>• Children autonomously seek and enjoy physical contact to others, while recognizing and respecting their boundaries<br>•Children have joy and stamina to master difficult movement-related tasks with others	• Children learn that repetition is a method that can develop physical skills<br>• Children learn not to be dissuaded from failures and to try again<br>• Children develop an awareness of their own abilities and potential for growth, and gradually increase the level of difficulty<br>• Children learn to recognize self-efficacy, participation and involvement as the cause of mental and physical well-being<br>• Children develop a desire to learn and are curiosity for further experiences and want to expand the area in which they are active, and one's own evaluation competence

**HESSE**	•Children develop a positive self-perception regarding physical attractiveness and athletic performance through movement<br>• Children increase self-esteem through greater movement-confidence<br>• Children recognice movement as an opportunity to increase positive emotions, inner balance and to control impulses.<br>• Children experience relaxation and tension<br>• Children can realistically assess personal performance<br>• Children develop joy of movement and a willingness to be active<br>• Children can identify individual progress in performance<br>• Children develop curiosity for new movement patterns and challenging motor skills<br>• Children developing body awareness and body consciousness	• Children recognize individual movement needs and gain movement experience<br>• Children test and develop motor and coordination skills<br>• Children develop conditional skills<br>• Children identify their physical limits and expand them through practice<br>• Children learn the basics of back-friendly behavior<br>• Children learn how to properly use play objects and sports equipment, and various technical terms used in sports	• Children develop a team spirit and learn to cooperate with others in movement tasks<br>• Children experience joy from moving with others and learn to adhere to rules<br>• Children practice to be considerate of others and to be fair<br>• Children experience movement as form of interaction and communication	• Children stengthen their ability to concentrate on certain movement patterns<br>• Children develop imagination and creativity by trying new movement ideas<br>• Children learn to understand connections between exercise, nutrition and health
**MECKLENBURG-WESTERN-POMERANIA**	• Children get to know their own body its abilities and limits,<br>• Children develop an awareness of their own motor skills<br>• Children develop an individual need for movement<br>• Children develop the need to move, diverse and engaging<br>• Children develop the need to explore and try out new movement opportunities.<br>• Children develop the need to explore the environment with all senses,<br>• Children develop the need to imitate movements of others<br>• Children develop the need to show their improved performance and compare themselves to others	• Children notice and controll their body in a variety of movement opportunities and play areas involving all the senses<br>• Children learn and apply basic movement skills<br>• Children develop physical skills<br>• Children develop fine-motor skills<br>• Children learn rules of movement and sports games and can apply them<br>• Children learn to perform combinations of running/jumping and throwing/catching<br>• Children can independently implement a play idea using different materials and objects.	• Children learn fair interaction during physical activity, and to pay attention to agreed rules<br>•Children can understand and enact the dual role of "together-against each other" in play, demonstrating solidarity and fair behavior.<br>• Children can develop their own play ideas and help shape play situations within a group.	**n/a**
**LOWER SAXONY**[1]	• Children develop a body awareness in a holistic sense and enhhance hearing ability	• Children can use their strength and can control their movement	**n/a**	**n/a**
**NORTH RHINE WESTPHALIA**[1]	• Children independently search for movement possibilities and fine and gross motor challenges, e.g.:<br>• Children can experience success, regardless of their state of motor-skills<br>• Children can be creative and build opportunities for movement in the environment<br>• Children experience a balanced ratio of tension and relaxation	• Children move and test their body outside the weekly exercise program or gym class/physical education andd have a variety of daily movement experiences according to their age and development<br>• Children experience their individual physical limits<br>• Children can try things out and make movement experiences without being held back by overly anxious adults<br>• Children develop fine motor skills in everyday situations at the own pace<br>• Children try different means of transportation and learn how to use them<br>• Children get used to the element of water and learn how to move in it	• Children independently search for movement opportunities and fine- and gross-motor challenges:<br>• Children learn to recognise and accept the physical skills of others	• Children independently search for movement possibilities and fine and gross motor challenges
**RHINELAND PALATINATE**[1]	• Children can feel natural joy of movement and are supported in their individual level of activity<br>• Children are enabled to experience the importance of physical activity for their physical well-being<br>• Children are being sutainably strengthened regading their natural joy of movement<br>• Children develop courage to use their motorskills	**n/a**	**n/a**	**n/a**
**SAARLAND**	• Children enjoy movement and explore their physical skills<br>• Children develop an awareness that their individual well-being can be controlled through movement	• Children aquire physical dexterity, flexibility, coordination skill, and an interest in physical activities<br>• Children enjoy and have the stamina to master difficult movement-related challenges with others	• Children experience joy from moving with others<br>• Children can contribute suggestions and solutions to movement-related games and can represent their own interests towards others, can accept the rules of the play group and are able to cooperate<br>• Children are able to accept challenges from others and can set boundaries	• Children gain a basic unterstanding about the different physical abilities of children in their group<br>• Children develop a desire to learn and to be curious for further experiences and the expansion of one's own area in which they are active
**SAXONY**[1]	• Children can satisfy the natural need for movement<br>• Children get to know their own body throughh movement<br>• Children can express emotions though their movement<br>• Children can reduce and channel aggression throug movement<br>• Children's well-being is improved by positive movement experiences<br>• Children develop a body-awareness<br>• Children can explore the environment through movement<br>• Children move freely based out of their own initiative<br>• Children practice movement patterns independently<br>• Children allow time for rest and relaxation	• Children develop a desire to move<br>• Children develop physical and motor competences<br>• Children explore their environment and acquire technical skills through movement-related games	• Children acquire social skills through p activity<br>• Children engage in social interaction via movement<br>• Children learn to communicate through movement in the early childhood	**n/a**
**SAXONY-ANHALT**[1]	• Children learn that bodily movement can actively create well-being.<br>• Children work with their bodies and explore their limits<br>• Children repeat and refine movement patterns	• Children refine movement patterns	**n/a**	**n/a**
**SCHLESWIG-HOLSTEIN**[1]	• Children learn to receive important feedback about themselves through body awareness which forms the basis for a sensitive approach to their own health	• Children actively search for gross- and fine-motor movement opportunities in indoor and outdoor spaces	**n/a**	• Children understand that learning and movement are one unit

**THURINGIA**[1]	• Children increase well-being though stimulating their senses and experiences reactions of their body.<br>• Children develop joy of movement<br>• Children freely develop individual movement-skills<br>• Children intuitively seek opportunities in which physical and gravitational sensations can be combined with visual and auditory perceptions<br>• Children develop a concept on which behaviors are beneficial to health	• Children develop the interaction between the postural motor system and directed motility as well as between gross- and fine-motor skills<br>• Children refine mimetic/ imitative skills in movement play<br>• Children learn movement skills such as swimming and cycling while interacting with caregivers<br>• Children learn to observe and imitate everyday- and working movements, gender-specific movement stereotypes, and their relativization, as well as sports, and dance movements	• Children learn that their independence is respected<br>• Children explore their body/ and conquer the world via motor skills in a protective bond with their caregiver without experiencing restriction<br>• Children develop an independent behaviour early on due to reliable relationships.<br>• Children learn to be considerate of the physical well-being and movement movement expressions of others<br>• Children adopt the movement skills and movement ideas of others and contribute their own movement ideas<br>• Children use opportunities to imitate/ talk about movements	• Children learn to improvise or develop movement patterns through imitation or creativity to express their needs, dreams and wishes

[1] Competences were assigned by the research team

[2] Additional exercise-specific goals:
Compensating for lack of exercise, strengthening the postural apparatus, developing efficient organs, increasing physical and mental well-being, perceiving movement as an opportunity to express one's feelings and to strengthen impulse control, and inner balance.

Legend: n/a: Information not available
