# Supplementary material for: Physical activity promotion in the early childcare setting: a content analysis of the federal-state-wide educational framework plans in Germany
Source: BMC Public Health. 2025 Aug 14;25:2759. doi: 10.1186/s12889-025-23798-7 (PMC12351901; doi:10.1186/s12889-025-23798-7)
Supplement: Supplementary file 5 — Additional file 5. Detailed overview of political measures for physical activity promotion [file 12889_2025_23798_MOESM5_ESM.pdf]

Additional file 5. Detailed overview of political measures for physical activity promotion

Federal state	Structural environment		Multipliers	
	Building (indoor/outdoor)	Suggested Material	Staff	General suggestions
**BADEN-WUERTTEMBERG**			**n/a**	
**BAVARIA**	• ECC has a gym or multipurpose-room that can be freely accessed every day<br>• Siderooms, corridors or corners are designed as movement-areas<br>• Outdoor area can be freely accessed and is designed in an activity-friendly way<br>• ECC area includes slopig areas, stairs, ladders and platforms for children tocrawl, climb or step-up	• Soft floor mats for jumping, rolling and tumble<br>• Foamed material<br>• Skipping ropes<br>• Parachute<br>• Various balls<br>• Everyday Material (e.g. toilet rolls)<br>• Pedalo®<br>• Skateboards<br>• Slow Motion Balls<br>• Balance boards<br>• Ropes to pull up or swing,<br>• Tunnels<br>• Hoops	• Staff allow children to use available movement opportunities and provide freedom for independent experimenting	• Involvement of parents during specific activities<br>• Providing parent-teacher conference on the topic of "play and exercise"<br>• Cooperating with local sport clubs<br>• ECC offer open exercise programs<br>• ECC offer structured exercise lessons for children over the age of 3<br>• ECC use the direct surrounding that can be used for activity (e.g. forest, maedows, parks or other open areas, or visits to the swimmingpool)
**BERLIN**	• Designing Spaces for Movement, e.g. having spacious outdoor areas for diverse movements, alternatively daily use of public green spaces and playgrounds, or frequent visits to Berlin's forests<br>• Activity-friendly design of interior, e.g. Development-appropriate furniture that stimulates movement and learning; available space for large-scale, noisy movements, use of corridors and entrance areas for playing and running<br>• Activity-friendly design of outdoor spaces, e.g. Places for climbing, jumping, slopes; places with different heights<br>• The interior design and the outdoor area<br>- Offer protected play areas that can be used independently<br>- Support movement activity with a sufficient number of stimulating materials for pushing, riding and climbing<br>- Encourage children to engage in a variety of movement activities	• Tricycles<br>• Balance bikes<br>• Skateboards<br>• Trampolines<br>• Skipping ropes<br>• Balls<br>• Climbing walls<br>• Balancing opportunities<br>• Tunnel<br>• Big boxes<br>• Dedicated movement area ("Bewegungsbaustelle")	• Staff encourage children to experience a variety of gross and fine motor movements in a group<br>• Staff support children in the expansion of their area in which they play and move<br>• Staff support children in developing a positive relationship to their body and its capabilities<br>• Staff are being a role model for the joy of movement<br>• Staff provide the youngest children with a wide range of sensory experiences through a flexible and stimulating space	• Weekly/frequent forest days are incorporated in the schedule<br>• ECC enable a variety of movement experiences<br>• Clarification/observation of the child's/family's movement behavior and the childs environment:<br>- Preferred types of movement? Movement games?<br>- Relaxation through movement or rest?<br>- Importance of exercise/sport in the family?<br>- Opportunities for exercise in the home, on playgrounds in the neighborhood?<br>• ECC provide appropriate support for children with mobility impairments<br>• ECC alternate phases of activity/movement and relaxation/rest<br>• ECC offer open exercise programs<br>• ECC encourage movement in the group<br>• Games that promote body awareness by full-body movements are being played
**BRANDENBURG**	• Places to romp around<br>• Places to climb<br>• Wide areas in group rooms and corridors without furniture<br>• Larger and smaller slopes<br>• Various stairs<br>• Different levels<br>• Retreats to relax	• Skateboards<br>• Trampoline<br>• Pedalo®<br>• Ropes<br>• Balls<br>• Climbing wall<br>• Vehicles<br>• Hammocks<br>• Opportunities for balancing	• Staff keep up to date with the current pedagogical discussions regarding the support and challenge of movement competence<br>• Staff create situations that allow children to experience their own bodies and offers both guided and free exercises so that the child can vary, experiment and challenge themselves<br>• Staff encourage and support children to develop healthy habits<br>• Staff spends lots of time with the children in the fresh air and moving around.<br>• Staff makes the facility movement-friendly.Clear away chairs and tables where they are not reallyare not really needed and make sure that the rules that apply in the daycare center do not inhibit movement. Do not make the children sit down if this is not absolutely necessary.<br>• Staff establishes movement rituals and movement breaks<br>• Staff gives impulses for movements of varying intensity, including daily activities that are strenuous, make the heart beat faster and make them sweat.<br>• Staff makes sure there are enough suitable objects that children can use for exploratory and cooperative movement games and that are suitable for pushing, rolling, sliding, balancing and climbing,<br>• Staff provides enough suitable objects that children can use for exploratory and cooperative movement games and that are suitable for pushing, rolling, sliding, balancing and climbing, for example. Place tree trunks, ribbons, ropes or balancing poles balancing poles in your playground and use as many natural materials as possible.<br>• Staff encourages even young children to climb stairs themselves, master inclined surfaces on foot and with bobby cars, climb, throw and catch balls, run, jump and hop.	• Psychomotor activity should be permanently incorporated in the program of a ECC<br>• ECC incorporate movement games

**BREMEN**	• Existence of a place where children can pursue their writing, painting or building activities undisturbed, and were they can interrupt and resume their activity<br>• The rooms and outdoor area are designed to enable and challenge different forms of movement<br>• ECC offer stimulating indoor and outdoor exercise areas, a variety of materials that encourage movement games and time for unrestricted exercise	• Availability of diverse play equipment for children of all developmental levels	• Staff create opportunities for the child to cross the center line of the body	• Regular exercise programs are provided throughout the day<br>• A variety of exercise options at different times and in different places, e.g. by visiting exercise areas outside the kindergarten, if possible both in urban areas and in natural surroundings<br>• Children have constant access to equipment and materials that promote fine motor skills<br>• Movement activities that stimulate the three basic areas of perception<br>- For balance: swinging, rocking, turning, rolling - all spatial directions can be experienced starting from the body<br>- For the muscles and joints: climbing, hanging, jumping, crawling, balancing, hiding, building, constructing movement construction sites<br>- For the sense of touch: Provide various materials for feeling, building, hiding, cuddling<br>• The children are being offered movement games in which they learn about rules, adaptation, movement control and social skills<br>• Movement and learning are inextricably linked, rooms should allow a variety of movement options<br>• Physical movement as the basis of all development, e.g:<br>- Play combines imagination and movement: The specialist provides the child with play equipment for large-scale movements<br>- Play combines imagination and movement: The specialist introduces simple group games<br>• Promoting movement in a holistic movement approach through music, language and movement<br>- Songs/music should encourage play and (rhythmic) movement<br>- Turning music into movement: Creating situations to move to music, to learn dances, introducing movement games using music
**HAMBURG**	• Indoor and outdoor spaces are designed so there is room for the younger and for the older children to retreat and rest, as well as sufficient space for a variety of physical activities and experiences of nature<br>• Rooms and outdoor areas are designed with a variety of movement options and challenges that children can use and accept independently<br>• Equipment of the outdoor area enables a variety of perceptual and movement experiences<br>• ECC offer developmentally appropriate furniture for children and adults; an environment that stimulates movement and learning for the different age groups	Play materials and play stimulation: Equipment and materials for children of all ages and stages of development<br>• Tricycles<br>• Balance bikes<br>• Skateboards<br>• Trampolines<br>• Ropes<br>• Balls<br>• Climbing wallds<br>• Balancing opportunities<br>• Tunnel<br>• Big boxes<br>• Dedicated movement area ("Bewegungsbaustelle")	• Staff support the fun and enjoyment of physical activity and promote the development of physical and motor skills<br>• Staff support physical activity by providing stimulating materials and encouraging children to engage in a variety of physical activities<br>• Staff create individual movement stimuli for individual children and addressing movement skills<br>• Staff are being a role model for the joy of movement and healthy activity	• ECC create conditions for alternating between activity/relaxation, rest/movement, while ensuring a good balance of movement and rest phases<br>• Exploring new movement stimuli in playgrounds and other places in the wider environments<br>• ECC revive traditional games (e.g. Tag, jumping games)
**HESSE**			**n/a**	
**MECKLENBURG-WESTERN-POMERANIA**			**n/a**	
**LOWER SAXONY**	• Wide areas without furniture, various levels, sloping surfaces, stairs and ladders<br>• ECC encourage movement through open spaces	• Physical dexterity is being encouraged through a wide range of equipment and materials<br>• Rocking, swinging, sliding, climbing, balancing, jumping and moving around is made possible by rolling and driving equipment<br>• Ropes<br>• Balls<br>• Pedalo®<br>• Vehicles<br>• Skateboards<br>• Trampolines<br>• Hammocks<br>• Swings	• Staff encourage and challenge children and offer wrestling, boxing, fencing according to jointly developed rules<br>• Staff pay particular attention to ensuring that both genders can experience equal oppurtunities, e.g., boys are encouraged to develop body awareness or fine motor skills, and girls are encouraged to try "wild" forms of movement	• Targeted small group support in a playful way, taking into account the children's individual stage of development and needs<br><br>• Endurance can be improved by running and catching games<br><br>• Children are encouraged to move to sounds or songs, to adjust to tempo, tone and rhythm, and to invent and create own forms of movement and dance.<br><br>• ECC can use a gym, visit an outdoor or indoor swimming pool or spend time in a park, maedow or forest
**NORTH RHINE WESTPHALIA**	• Room-design takes into account the children's urge to move<br>• The entire environment should be designed to be movement-friendly so all children can pursue their joy of movement according to their individual abilities	• Different play equipment and materials, means of transportation and terrain experiences<br>• Vehicles, e.g., tricycle, balance bike, scooter, bike<br>• For children in the first years of life: The equipment in the rooms and the play and activity materials are age-appropriate and appropriate opportunities for movement are provided	**n/a**	• ECC should pay specific attention to potential movement-restrictions in everyday life:<br>- Unnecessary rules<br>- Overly cautious reactions and actions by professionals<br>- Too much furniture<br>- Limited movement times<br>- Lack of opportunities for outdoor experiences<br><br>• ECC offer (sporty) games<br>• In the ECC movement education is part of daily routines and is linked to all other educational areas
**RHINELAND PALATINATE**	• The outdoor area is designed to facilitate the experience of nature and movement for all age groups	**n/a**	• Staff should frequently observe the physical activity behavior of children	• Cooperation with sports clubs and specialist services is encouraged<br>• ECC enable children to:<br>- Try out and practicing different types of movement such as balancing, climbing, running, jumping etc. at any time<br>- Discover and try new movements<br>- To use the indoor and outdoor spaces of ECC for sports, movement games and spontaneous movements at any time

**SAARLAND**	• Rooms and outdoor areas are designed with a variety of movement opportunities and challenges, which individual children can independently use<br><br>• Rooms and outdoor areas:<br>- Facilitate the experience of sun, wind, rain<br>- Include cooling and warming materials<br>- Include water, splash and mud areas, retreat and relaxation corners, sensory rooms (snoezelen), places to climb, places to romp sensory trails - feel and touch routes, height differences indoors and outdoors, mirror worlds, movement construction sites	• Movement materials that each individual child can use independently:<br>- Skateboards<br>- Trampolines<br>- Ropes<br>- Balls<br>- Climbing walls<br>- Balancing opportunities<br>- Vehicles	• Staff adress individual physical activity competencies and encourage children<br>• Staff observe the childrens' needs for movement and rest, for physical discomfort and well-being	• ECC offer movement adventures<br>• ECC offer frequent forest days<br>• ECC educate children on movement possibilities in summer/winter<br>• ECC revive traditional games (e.g. Tag, jumping games)
**SAXONY**	• ECC should include sufficient areas for movementand offer specific movement-friendly material, e.g. platforms, climbing scaffolds, caves, and movable or stackable materials<br>• ECC offer rest and retreat areas for independent use	**n/a**	**n/a**	• ECC include movement as a direct and indirect goal in other areas of education (somatic, communicative, aesthetic and scientific education)<br>• Supplementing freely accessible offers with movement education activities for sensory experience, motor skills, coordination
**SAXONY-ANHALT**	• Rooms are designed according to childrens' needs and encourage movement rather than restrict it and create stimuli in all dimensions of space.<br>• Outdoor spaces use natural materials, e.g., sand, stone, wood or water to encourage movement<br>• Outdoor spaces encourage movement through its design, e.g. platforms, hills, ditches, inclines, paths and areas for fast, extensive movement	**n/a**	• Staff support movement in all eductional processes at any time.<br>• Staff support motor development through a variety of movement stimuli, and encourage children to make their first attempts at crawling, standing and walking and trust them when they are climbing and trying out the stairs<br>• Staff recognize which interests and topics each child pursues in connection with their body through systematic and resource-oriented observation<br>• Staff do not impede childrens' natural urge to move<br>• Staff encourage movement through the design of the rooms, provided materials and being a role model, rather than through guided physical activity programs<br>• Staff identify reasons for inactivity, if a child frequently evades physical challenges, and communicates with the child, the team and the parents<br>• Staff ensure that children can move according to their needs in every situation	• ECC use the surrouding social areas (e.g., forests, parks and meadows) to encourage movement<br>• ECC acknowledge movement as a prerequisite for successful educational processes<br>• Children have access to all rooms at all times, including the outdoor area and are allowed to go there according to their needs. Rules are established for this in a participatory manner<br>• ECC cooperate with sport clubs, if the offer can be used by all children
**SCHLESWIG-HOLSTEIN**	• The indoor areas or rooms have different levels that are not blocked by tables and chairs, that encourage children to move; plateaus for climbing and taking stairs; slopes for rolling and sliding; a glued on line to encourage balancing<br>• The outdoor area includes naturally designed outdoor areas with hills, climbing trees and play areas encourage a variety of movement sequences; bushes for hiding; hills and meadows; sand with water; tunnels to crawl; tents; hard surface pitch for vehicles; tipped over tree	• Hammocks<br>• Rope ladder<br>• Therapy swings<br>• Vehicles and skateboards<br>• Balls	• Staff provide children with a variety of stimuli for movement and sensory experiences in both indoor and outdoor spaces.<br>• Staff support children with impairments based on their individual needs	• ECC implement movement, e.g. through ball games, running and catching games, skill and reaction games, etc.
**THURINGIA**	• Indoor and outdoor areas are designed in a movement-friendly way including:<br>- equipment that encourages movement<br>- versatile and movement-friendly furnishings<br>- height adjusted chairs and play furniture items<br><br>• ECC offer areas to walk barefoot in nature or indoors, and to walk on different surfaces; they offer opportunities to play on swings, rockers, carussels, balance boards; to experience swimming, riding a bik, singing and dancing	• Tricycles<br>• Balance bikes<br>• Skateboards<br>• Trampolines<br>• Ropes<br>• Balls<br>• Climbing walls<br>• Balancing opportunities	• Staff provide momevent opportunities, that foster children's individual experiences, and take into account their perception and wishes<br>• Staff offer various situations that present new challenges and in which children have to prove themself with effort<br>• Staff offer space for children's own ideas for movement and play, rules are negotiated in consultation with partners and the group<br>• Staff support and accompany a wide range of movement activities that are sought out by the children themselves<br>• Staff observe the childrens' urge to explore without inappropriate, hasty intervention to allow the child's body sensory and movement-oriented needs to be expressed<br>• Staff identify the need for rest and movement and respect both	• ECC can cooperate with sportclubs<br>• ECC use everyday life, dream- or fantasyworld as a reason for movement games or roleplays<br>• ECC enable the participation in the planning of movement-related projects (e.g. dance-performance during festivities<br>• Physical activity is included in other educational areas (e.g., mathematics)<br>•ECC stimulate and facilitate the experience of interplay between breathing, posture, support-, aim-, gross- and fine motor skills in many situations<br>•ECC offer balancing games, running and catching games, running in circles with centrifugal and gravitational forces

Legend: n/a: Information not available

Abbreviations: ECC Early childcare center
